# Supplementary material for: Neurotrauma clinicians’ perspectives on the contextual challenges associated with long-term follow-up following traumatic brain injury in low-income and middle-income countries: a qualitative study protocol
Source: BMJ Open. 2021 Mar 4;11(3):e041442. doi: 10.1136/bmjopen-2020-041442 (PMC7934765; doi:10.1136/bmjopen-2020-041442)
Supplement: Author's manuscript [file bmjopen-2020-041442.draft_revisions.pdf]

BMJ Open is committed to open peer review. As part of this commitment we make the peer review history of every article we publish publicly available.

When an article is published we post the peer reviewers' comments and the authors' responses online. We also post the versions of the paper that were used during peer review. These are the versions that the peer review comments apply to.

The versions of the paper that follow are the versions that were submitted during the peer review process. They are not the versions of record or the final published versions. They should not be cited or distributed as the published version of this manuscript.

BMJ Open is an open access journal and the full, final, typeset and author-corrected version of record of the manuscript is available on our site with no access controls, subscription charges or pay-per-view fees (http://bmjopen.bmj.com).

If you have any questions on BMJ Open's open peer review process please email info.bmjopen@bmj.com

BMJ Open

Contextual challenges associated with Long-term follow-up following Traumatic Brain Injury in Low- and Middle-income countries: a qualitative study protocol

Journal:	*BMJ Open*
Manuscript ID	bmjopen-2020-041442
Article Type:	Protocol
Date Submitted by the Author:	09-Jun-2020

Complete List of Authors:	Smith, Brandon; Addenbrooke's Hospital & University of Cambridge, Division of Neurosurgery, Department of Clinical Neurosciences; University of Cambridge, NIHR Global Health Research Group on Neurotrauma<br>Whiffin, Charlotte; University of Derby, College of Health of Social Care; University of Cambridge, NIHR Global Health Research Group on Neurotrauma<br>Esene, Ignatius ; University of Bamenda, Cameroon, Neurosurgery Division, Faculty of Health Sciences,<br>Karekezi, Claire; Rwanda Military Hospital, Department of Neurosurgery<br>Bashford, Tom; University of Cambridge, NIHR Global Health Research Group on Neurotrauma<br>Mukhtar Khan, Muhammad; Northwest School of Medicine and Northwest General Hospital and Research Centre, Neurosurgery; University of Cambridge, NIHR Global Health Research Group on Neurotrauma<br>Fontoura Solla, Davi; University of São Paulo Department of Neurosciences and Behaviour Sciences; University of Cambridge, NIHR Global Health Research Group on Neurotrauma<br>Indira Devi, Bhagavatula; National Institute of Mental Health and Neuro Sciences; University of Cambridge, NIHR Global Health Research Group on Neurotrauma<br>Hutchinson, Peter; Addenbrooke's Hospital & University of Cambridge, Division of Neurosurgery, Department of Clinical Neurosciences; University of Cambridge, NIHR Global Health Research Group on Neurotrauma<br>Kolias, Angelos; Addenbrooke's Hospital, University of Cambridge, Division of Neurosurgery, Department of Clinical Neurosciences; University of Cambridge, NIHR Global Health Research Group on Neurotrauma<br>Figaji, Anthony; University of Cape Town; University of Cambridge, NIHR Global Health Research Group on Neurotrauma<br>Rubiano, Andres M; Universidad El Bosque, Neurosciences Institute, Department of Neurosurgery; University of Cambridge, NIHR Global Health Research Group on Neurotrauma

Keywords:	QUALITATIVE RESEARCH, NEUROSURGERY, Neurological injury < NEUROLOGY

I, the Submitting Author has the right to grant and does grant on behalf of all authors of the Work (as defined in the below author licence), an exclusive licence and/or a non-exclusive licence for contributions from authors who are: i) UK Crown employees; ii) where BMJ has agreed a CC-BY licence shall apply, and/or iii) in accordance with the terms applicable for US Federal Government officers or employees acting as part of their official duties; on a worldwide, perpetual, irrevocable, royalty-free basis to BMJ Publishing Group Ltd ("BMJ") its licensees and where the relevant Journal is co-owned by BMJ to the co-owners of the Journal, to publish the Work in this journal and any other BMJ products and to exploit all rights, as set out in our licence.

The Submitting Author accepts and understands that any supply made under these terms is made by BMJ to the Submitting Author unless you are acting as an employee on behalf of your employer or a postgraduate student of an affiliated institution which is paying any applicable article publishing charge ("APC") for Open Access articles. Where the Submitting Author wishes to make the Work available on an Open Access basis (and intends to pay the relevant APC), the terms of reuse of such Open Access shall be governed by a Creative Commons licence – details of these licences and which Creative Commons licence will apply to this Work are set out in our licence referred to above.

Other than as permitted in any relevant BMJ Author's Self Archiving Policies, I confirm this Work has not been accepted for publication elsewhere, is not being considered for publication elsewhere and does not duplicate material already published. I confirm all authors consent to publication of this Work and authorise the granting of this licence.

For peer review only

Title page

Full title: Contextual challenges associated with Long-term follow-up following Traumatic Brain Injury in Low- and Middle-income countries: a qualitative study protocol
Short title: Long-term follow-up of TBI in LMICs

Smith BG* [1 2], Whiffin CJ* [1 2 3], Esene IN[4], Karekezi C[5], Bashford T[1 2], Khan M[2 6], Solla DJF[2 7], Devi BI[2 8], Hutchinson PJ[1 2], Kolias AG[1 2], Figaji AA[2 9], Rubiano AM[2 10]

* joint first authors

[1] Division of Neurosurgery, Department of Clinical Neurosciences, Addenbrooke's Hospital and University of Cambridge, Cambridge, U.K.
[2] NIHR Global Health Research Group on Neurotrauma, University of Cambridge, Cambridge, UK
[3] College of Health and Social Care, University of Derby, U.K.
[4] Neurosurgery Division, Faculty of Health Sciences, University of Bamenda, Cameroon
[5] Rwanda Military Hospital, Department of Neurosurgery, Rwanda
[6] Northwest School of Medicine & Northwest general Hospital & Research Centre, Peshawar, Pakistan
[7] University of São Paulo, Brazil
[8] Department of Neurosurgery, NIMHANS, Bangalore, India
[9] University of Cape Town, Cape Town, South Africa
[10] Institute of Neurosciences and Neurosurgery, El Bosque University, Bogotá, Colombia

Corresponding author
Angelos G. Kolias
Clinical Lecturer in Neurosurgery
Division of Neurosurgery
Box 167
University of Cambridge
Cambridge Biomedical Campus
Cambridge, CB2 0QQ,
United Kingdom

Email address: ak721@cam.ac.uk

For peer review only

ABSTRACT

Introduction

Traumatic Brain Injury is a global public health concern; however, low- and middle-income countries (LMICs) face the greatest burden. The World Health Organisation recognise the significant differences between patient outcomes following injuries in high-income countries versus those in LMICs. Outcome data is not reliably recorded in LMICs and despite improved injury surveillance data, data on disability and long-term functional outcomes remains poorly recorded. Therefore, the full picture of outcome post-TBI in LMICs is largely unknown.

Methods and Analysis

This is a cross-sectional pragmatic qualitative study using individual semi-structured interviews with physicians who have experience of neurotrauma in LMICs. The aim of this study is to understand the contextual challenges associated with long-term follow-up of patients following traumatic brain injury in LMICs. For the purpose of the study we define 'Long-term' as any data collected following discharge from hospital. We aim to conduct individual semi-structured interviews with 24 to 48 neurosurgeons. Interviews will be recorded and transcribed verbatim. A thematic analysis will be conducted supported by NVIVO software.

Ethics and Dissemination

The University of Cambridge Psychology Research Ethics Committee approved this study in February 2020. Ethical issues within this study include consent, confidentiality and anonymity, and data protection. Participants will provide informed consent and their contributions will be kept confidential. Participants will be free to withdraw at any time without penalty; however, their interview data can only be withdrawn up to one week after data collection. Findings generated from the study will be shared with relevant stakeholders such as the World Federation of Neurosurgical Societies and disseminated in conference presentations and journal publications.

Registration*: Not applicable.*

ARTICLE SUMMARY

Study Strengths and Limitations

- This study is among the first to examine the contextual challenges associated with long-term follow-up of patients following traumatic brain injury in LMICs.
- Findings generated from this study will enable us to establish an understanding of the ways long-term follow-up of neurotrauma outcomes can be successfully recorded in LMICs, and may facilitate the ongoing development of cost effective, novel follow-up technologies.
- Through our non-probability sampling technique and necessity for a small sample size, not all LMICs will be represented in this study, however an economic and geographic spread is sought.
- Due to the resources available and the need to conduct in-depth interviews and subsequent qualitative analysis, it was not possible to recruit non-English speaking physicians.

Key Words:

- Traumatic Brain Injury
- Qualitative Research
- Global Neurosurgery
- Follow-up

Word count: 2938

For peer review only

INTRODUCTION

Background

Traumatic Brain Injury (TBI) is a global public health concern; however, low- and middle-income countries (LMICs) face the greatest burden[1]. The World Health Organisation recognise the significant differences between patient outcomes following injuries in high-income countries (HICs) versus those in LMICs[2]. In high-income countries improvements in surgical interventions and medical management has meant rapid increases in survival rates and improved outcomes. In LMICs fewer resources, different treatment practices and reduced availability of surgical interventions has meant these countries have not all seen the same improvements in outcome post-TBI[3].

Acknowledging heterogeneity within both LMICs and HICs, as a whole outcome data is generally not reliably recorded in LMICs, and despite improved injury surveillance data, data on disability and long-term functional outcomes remains poorly recorded[4]. Therefore, the full picture of outcome post-TBI in LMICs is largely unknown. Laytin et al (2018) described the difficulty of collecting outcome data in resource poor settings, specifically Sub-Saharan Africa. Collection of outcome data in these settings was typically conducted within the emergency department or at hospital discharge rather than at points after discharge. Challenges of collecting outcome data included weak healthcare infrastructure and limited regular follow-up of trauma patients[4].

Despite such challenges the collection of long-term outcomes is essential, as it provides epidemiological data to properly determine the full burden of injury and assess the efficacy of patient treatment and management decisions in LMICs[4,5] In addition Mock et al.,[2] argues that to fully understand the full burden of disease, disability must be evaluated as well as death. The collection of long-term outcomes is additionally important in the implementation and continuation of registries. These resources serve to provide opportunities for continuous quality improvement and enable trials of interventions in these populations – the results of these registries can themselves be incorporated into legislation and specific care pathways[6]. Therefore, ways must be found to collect more complex long-term outcomes following TBI in LMICs that are directly comparable to the outcome data being collected in most high-income countries. In turn, we must be sensitive to the economic capacities of each country versus their quality of follow-up provision, and acknowledge that the distinction between countries by economic brackets is somewhat artificial; that the gross national income per capita does not necessarily follow the efforts and successes being made by neurosurgical departments, so as to not further encourage any existing false dichotomies.

Rationale

Very little is published in the empirical literature about the difficulties long-term follow-up poses within LMICs and what specific contextual challenges exist within these countries. Without an in-depth understanding of these contextual challenges it is not possible to make recommendations or suggest ways to improve the collection of long-term outcomes in these countries. There is a growing notion in literature that a synergy exists between the utilisation of trauma registries, quality improvement programmes and long-term outcomes, and have all been advocated into systems science approaches for improving neurotrauma care in all regions, especially in LMICs where such registries and schemes are largely absent[6–8]. We wanted to explore this problem in full first using a qualitative design as part of the NIHR Global Health Group on Neurotrauma. The NIHR Global Health Group on Neurotrauma commenced in 2017 aiming to understand current systems and challenges in TBI clinical care within LMICs and as part of this acknowledged that the collection of long-term outcomes needed to be improved. Although beyond the scope of this particular project, results of this study may warrant an investigation into how well long-term follow-up is conducted across the spectrum of high-income country neurosurgical centres, that are not directly conducting or engaging in long-term outcome studies for objective comparison. In addition, we will be careful to ensure our results are not compared to presumptions, or to centres with follow-up efforts motivated only by engagement in international studies.

Theoretical Framework

We propose a cross-sectional pragmatic qualitative study using individual semi-structured interviews with physicians who have experience of neurotrauma in LMICs. Qualitative inquiry is especially useful to explore complex phenomena[9], when little is known about a topic, or where new insights are required. Qualitative inquiry is committed to subjectivity and the collection of rich in-depth data through the examination of participant's viewpoints[9]. Pragmatic qualitative research, also known as descriptive and generic qualitative research, does not align itself to any specific philosophical perspective and therefore allows flexibility of methods that enables the study to be designed in an appropriate way to address the research question[10].

For peer review only

Pragmatic research lies within the naturalistic paradigm which recognises the existence of multiple realities and studies real-world situations by having little impact on the phenomena under investigation[11]. Pragmatic qualitative studies aim to achieve in-depth understanding prioritising literal description first and then understanding phenomena in a deeper sense through analysis and interpretation of how people draw meaning from their experiences[12]. The goal of such research is to provide an account of the "experiences, events and process that most people (researchers and participants) would agree are accurate"[13] p128. In addition, Sullivan-Bolyai, et al.[13] state qualitative descriptive designs are particularly useful when researching populations of other cultures and as such would be well suited to the aims of this study.

Research Aim

The aim of this study is to understand the contextual challenges associated with long-term follow-up of patients following traumatic brain injury in LMICs. For the purpose of the study we define 'Long-term' as any data collected following discharge from hospital.

Objectives

1. To determine current approaches to long-term follow up of patients following TBI in LMICs
2. To explore the challenges associated with long-term follow-up of patients following TBI in LMICs.
3. To collaboratively offer recommendations for appropriate solutions that will facilitate long-term follow-up of patients following TBI in LMICs based on findings and themes identified

METHODS AND ANALYSIS

Study Design

We will ask participants to take part in semi-structured telephone/online video interviews and provide relevant demographic and professional data.

Data collection

This study will collect data through semi-structured telephone or online video interviews. Semi-structured interviews are the cornerstone of qualitative methodology. Although face-to-face interviews are more common, the need to recruit participants from a wide range of different countries made this unfeasible. We will also collect some limited demographic data that will help us analyse and interpret the similarities and differences between the data sets (See Box One).

Specific Demographic data will be collected using an electronic form (see Box 1).

Age<br>Gender<br>Country of residence<br>Job title<br>Years of experience<br>Years working in neurotrauma<br>Practice setting
Box One: Demographic Data

Details of the interview questions are summarised in Box two, and participants will be made aware of these prior to the interview so they have the opportunity to consider their response. Questions are open ended to encourage a rich dialogue and participants will be free to elaborate on their answers adding additional detail as required. It is anticipated that the interviews will last up to 60 minutes and will be conducted by the UK research team following a pilot of the interview schedule. Interviews will be recorded using a digital recorder.

Current follow-up<br>A. Please can you tell me about how and when your patients are currently followed-up following discharge from a TBI?<br>a. What outcomes are measured?<br>b. What needs are assessed?<br>c. What referrals are made?

For peer review only

d. What type of contact system do you have in place (face-to-face, telephone, telemedicine)?

Definition and understanding of long-term outcomes

A. What do you understand by the use of 'long- term' in the context of outcomes post-TBI?
B. Do you think your definition and understanding of 'long-term outcomes' in your country are the same as those in other countries?
C. Do you know or use any type(s) of outcome classification for follow-up?

Attitudes towards long-term follow-up

A. What do you think about the need for long-term follow-up of patients following TBI in your setting?
B. What would you consider to be the main benefits of long-term follow-up of TBI patients?
C. What do you consider to be the challenges associated with long-term follow-up of your patients with TBI?
D. Are these challenges more related to: health system aspects, national, regional, or local administrative aspects, institutional aspects, resources for care aspects?
E. Are there any other issues that need to be considered for long-term follow-up in your own setting and wider country?

Long-term follow-up in LMIC

A. Thinking more broadly now about other Low and Middle Income Countries, do you have any other thoughts about long-term follow-up in these other LMICs
B. What else might be required in LMICs to facilitate long-term follow-up of patients following TBI?

Possible interventions to facilitate follow-up in LMIC

A. Do you know what types of technology patients and families have access to that could help in follow-up of patients following TBI?
B. What kind of technology would help in your country to record long-term follow-up?
C. Are you aware of any specific mobile health, telemedicine, or phone interview options for long-term follow-up in your institution/state/country?
D. Does your service or institution participate in a trauma or neurotrauma quality improvement program including the use of clinical registries with outcome measures?

Box Two: Interview schedule

Data Analysis

Analysis within pragmatic qualitative studies should stay close to the text, and does not require "highly abstract rendering of data"[12]. Studies which are independent of theory, can and often do use thematic analysis[9,14] According to Braun and Clarke[14] thematic analysis can reveal a rich, detailed and complex exploration of the data and despite its 'descriptive' title will end with some necessary interpretation of the data[12] through discovery of shared and varied patterns of understanding[15]. Indeed Vaismoradi, et al.[9] contends that thematic approaches actually attends little to the description of the data set and instead requires interpretation of the various aspects of the phenomena under investigation. It is therefore proposed that a six stage Braun and Clarke[14] thematic analysis will be conducted (See Box 3).

To complete the analysis audio files from interviews will first be transcribed verbatim by a transcription service and checked for accuracy by the research team. Codes will be assigned to individual transcripts and will be supported by the use of NVIVO software, allowing researchers to organise the data, share coding decisions and confirm the origins of interpretation. Similar codes will be grouped together, and initial themes identified. Themes will then be reviewed, revised and final themes named and agreed upon within the research team. BS will take lead on the data analysis, supported by CW in the initial coding stages. BS is a PhD student and fourth year medical student who has undertaken training in qualitative methods and analysis. CW is an experienced qualitative researcher and nurse academic.

1. Familiarising yourself with your data
2. Generate initial codes
3. Searching for themes
4. Reviewing themes
5. Defining and naming themes
6. Produce the report

Box Three: Braun and Clarke [14] thematic analysis framework

For peer review only

Rigour

Validity, generalizability and reliability have little relevance in qualitative research[16,17]. Instead qualitative research is judged by more appropriate criteria such as credibility and trustworthiness[18]. These hallmarks of quality determine the rigour of a qualitative study. In this study we intend to include a number of strategies to increase the rigour of the study and consequently its findings. Based on strategies used to increase rigour as suggested by Nicholls[18] analysis will involve discussion of emerging findings with other members of the research team. In addition, we propose using member checking as advocated by Lincoln and Guba[19] as crucial to establishing credibility in qualitative studies and according to Birt, et al.[20] can overcome issues of researcher bias and increases transferability of findings. In this regard, we will first return any transcripts where transcription was not possible due to the quality of recording to ask for clarification of specific sections of the interview if necessary. Second, we will share initial themes arising from the data with participants so they can and add further insight to the interpretation and emerging findings if they wish. These contributions will be added to the final analysis of data.

Study Setting

Participants will be recruited from any country identified as low or middle income as defined by the 2017-2018 World Bank list of economies[21]. Access to participants will be negotiated using the research group's project lead and collaborators from the twelve countries in the NIHR Global Health Research Group on Neurotrauma listed in Box Four. New collaborators in the group, including the Philippines and Zimbabwe, in addition to participants from institutions from LMICs not yet part of the group, will be invited to participate as necessary in order to attain the defined sample size.

1. BRAZIL	7. MYANMAR
2. COLOMBIA	8. NIGERIA
3. ETHIOPIA	9. PAKISTAN
4. INDIA	10. SOUTH AFRICA
5. INDONESIA	11. TANZANIA
6. MALAYSIA	12. ZAMBIA

Box Four: LMICs in the NIHR Global Health Research Group on Neurotrauma

Eligibility Criteria

To be eligible for this study the person must be a practising physician within an LMIC and have experience of managing neurotrauma, so they have experience of treatment and discharge of patients with TBI. We are requesting the physicians all have at least two years' experience of neurotrauma and also some experience of research, so they have more insight into the complexities of long-term follow-up of neurotrauma patients and understand the research process. Due to availability of resources participants will have to be fluent in English. Experience of working with collaborators would suggest this criterion will not limit participation in the study. All participants will have to provide informed consent by agreeing to the criteria within the consent form.

Inclusion criteria

- Physician within a LMIC with experience of managing neurotrauma
- At least two years' experience of neurotrauma
- Experience of collecting, or attempting to collect, TBI outcome data
- Self-declared fluency in spoken English
- Able to provide informed consent

Box Five: Inclusion criteria

Sample

The main criticism of qualitative studies stems from the necessity for small samples. Smaller samples facilitate depth of inquiry and rich interpretation. However, according to Sullivan-Bolyai, et al.[13] qualitative descriptive studies can accommodate more moderate sample sizes than theoretically informed studies because there is no requirement to interpret lived experiences or generate theory. Therefore Sullivan-Bolyai, et al.[13] recommends a sample size of 20-50 participants.

Moser and Korstjens[22] explain that a guiding principle of sampling in qualitative studies is to sample up until data saturation is reached. Data saturation is a qualitative principle which is a point whereby the researchers are confident that new data will not reveal new information[23]. Sample size in this study was further informed by desire to explore a variety of views from physicians within a range of LMICs. A non-probability, purposive sample is therefore required. We will aim to have representation from a range of countries and, if possible, a range of regions within these countries. Therefore we propose a sample size of 24 - 48 participants. Data collection will continue, within these parameters, until the research team are confident that saturation has been reached.

Recruitment

Collaborators in the NIHR Global Health Research Group on Neurotrauma include clinicians in 12 LMICs, with new collaborators from Zimbabwe and the Philippines. We will also be placing a call through social media to maximise the opportunity for recruitment.

The local PIs will be written to with information pertaining to the study including the aims of the study, inclusion criteria for participants and data collection methods. We will then ask the local PIs to forward information about the study to relevant physicians (NIHR GHRGN collaborators are also eligible to participate). Information will include a letter of invitation, participant information sheet, and contact details for the research team. If interested in participating, physicians can contact the research team via email. We will then arrange an initial telephone call to review the participant information sheet and the requirements of the study. If the physician still wishes to continue, we will schedule a telephone interview. We will leave a minimum of one week in-between this initial contact and arrangement of the interview to allow a cooling off period. If an insufficient number of people have responded to the first call for participants a second request for participation will be made two – three weeks later.

It is essential that participants join this study of their own free will. However, the notion that junior colleagues may have a 'reciprocal obligation' to assist their more senior colleagues, and the institution, in participating in global research is appreciated. To safeguard against potential coercion, we will highlight to our participants that they are under no obligation to take part. We will discuss this specifically during the pre-consent meeting and ensure they understand that no-one will be informed if they chose, or do not chose, to participate in the study.

ETHICS & DISSEMINATION

The University of Cambridge Psychology Research Ethics Committee reviewed this study with approval received in February 2020 (Ref: PRE.2020.010). The University of Cambridge is the sponsor and appropriate insurance is in place. Ethical issues within this study include consent, confidentiality and anonymity, and data protection. All participants will be required to provide informed consent after a cooling-off period of at least a week after the participant information sheet has been delivered. Participants can withdraw at any time without penalty, with a grace period of a week given after completion of the interview to withdraw their interview data, after which withdrawal of data will not be possible as analysis will have commenced.

All information will be kept strictly confidential, with all information generated to be stored on a GDPR compliant confidential server. A unique study ID will be allocated to all participants and used to anonymise personal data. Audio files will be immediately transferred to an encrypted folder and the data on the recording device destroyed, interview data will be anonymised upon transcription and other personal information changed or removed once checked by the research team. Participants will be told that anonymised quotes will be published in the findings of this study; however, despite this anonymity it is possible that their contributions may be recognisable to others reading the study. We will also be seeking consent to disclose region and level of income associated with their country of origin against participant quotes used in the publication of findings, as this will be important to the contextual understanding of the study.

Patient & Public Involvement

This study seeks to ascertain the views of physicians, and thus patient and public involvement was not considered to be as relevant in this context. However, we recognise the value of working with key stakeholders to develop research and therefore asked for peer review of this study by collaborating members of the GHRGN. Their comments informed the final study design.

For peer review only

Dissemination

When we have completed the study, we will produce a study summary which we will offer a copy to participants via email. Study findings will also be disseminated internationally through the GHRGN network and other relevant stakeholders such as the World Federation of Neurosurgical Societies, in addition to appropriate conferences, peer reviewed journal publications and social media.

Study Limitations

Understandably, through the non-probability approach to sampling and the necessity for a small sample size, not all LMICs will be represented in this study, however an economic and geographic spread is sought. We anticipate developing considerable insight from our international cohort of participants with differing levels of income and localities, in addition to establishing an understanding of the ways long-term follow-up of neurotrauma outcomes can be successfully recorded in LMICs. Careful attention to nuances must be paid. We respect and have the foresight that there will be heterogeneity within LMICs as a whole, and even within countries themselves as to the current provision, capabilities and attitudes towards neurotrauma follow-up. We must be sensitive when categorising our findings across a spectrum based on the gross national income per capita. We acknowledge that some false dichotomy may exist in the dialogue regarding healthcare provision, particularly of the collection of long-term outcomes, and caution will be exercised in interpretation of our findings. Unfortunately, due to the resources available and the need to conduct in-depth interviews and subsequent qualitative analysis, it was not possible to recruit non-English speaking physicians.

Notwithstanding, to our knowledge this study is among the first to examine the contextual challenges associated with long-term follow-up of patients following traumatic brain injury in LMICs. Findings generated from this study will enable us to establish an understanding of the ways long-term follow-up of neurotrauma outcomes can be successfully recorded in LMICs. This knowledge may lend itself to facilitating the ongoing development of cost-effective and novel follow-up technologies in the future.

References

1 Kolias AG, Rubiano AM, Figaji A, *et al.* Traumatic brain injury: global collaboration for a global challenge. *Lancet Neurol* 2019;18:136–7. doi:10.1016/S1474-4422(18)30494-0

2 Mock C, Lormand J, Goosen J, *et al.* Guidelines for essential trauma care. Geneva: 2004.

3 Servadei F, Tropeano MP, Spaggiari R, *et al.* Footprint of Reports From Low- and Low- to Middle-Income Countries in the Neurosurgical Data: A Study From 2015 to 2017. *World Neurosurg* 2019;130:e822–30. doi:10.1016/j.wneu.2019.06.230

4 Laytin AD, Seyoum N, Azazh A, *et al.* Feasibility of telephone-administered interviews to evaluate long-term outcomes of trauma patients in urban Ethiopia. *Trauma Surg Acute Care Open* 2018;3:e000256. doi:10.1136/tsaco-2018-000256

5 Laytin AD, Debebe F. The burden of injury in low-income and middle-income countries: knowing what we know, recognising what we don't know. *Emerg Med J* 2019;:emermed-2019-208514. doi:10.1136/emermed-2019-208514

6 Mowafi H, Ngaruiya C, O'Reilly G, *et al.* Emergency care surveillance and emergency care registries in low-income and middle-income countries: conceptual challenges and future directions for research. *BMJ Glob Heal* 2019;4:e001442. doi:10.1136/bmjgh-2019-001442

7 LaGrone LN, Romaní Pozo DA, Figueroa JF, *et al.* Status of trauma quality improvement programs in the Andean region: What foundation do we have to build on. *Injury* 2017;48:1985–93. doi:10.1016/j.injury.2017.03.003

8 Rubiano AM, Carney N, Chesnut R, *et al.* Global neurotrauma research challenges and opportunities. *Nature* 2015;527:S193–7. doi:10.1038/nature16035

9 Vaismoradi M, Turunen H, Bondas T. Content analysis and thematic analysis: Implications for conducting a qualitative descriptive study. *Nurs Health Sci* 2013;15:398–405. doi:10.1111/nhs.12048

10 Smith J, Bekker H, Cheater F. Theoretical versus pragmatic design in qualitative research. *Nurse Res* 2011;18:39–51. doi:10.7748/nr2011.01.18.2.39.c8283

11 Bowen GA. Naturalistic inquiry and the saturation concept: a research note. *Qual Res* 2008;8:137–52. doi:10.1177/1468794107085301

12 Bradshaw C, Atkinson S, Doody O. Employing a Qualitative Description Approach in Health Care Research. *Glob Qual Nurs Res* 2017;4:233339361774228. doi:10.1177/2333393617742282

13 Sullivan-Bolyai S, Bova C, Harper D. Developing and refining interventions in persons with health

For peer review only

disparities: The use of Qualitative Description. *Nurs Outlook* 2005;53:127–33. doi:10.1016/j.outlook.2005.03.005
14 Braun V, Clarke V. What can "thematic analysis" offer health and wellbeing researchers? *Int J Qual Stud Health Well-being* 2014;9:26152. doi:10.3402/qhw.v9.26152
15 Ulin PR, Robinson ET, Tolley EE, *et al. Qualitative Methods in Public Health: A Field Guide for Applied Research*. 2nd ed. 2016.
16 Korstjens I, Moser A. Series: Practical guidance to qualitative research. Part 4: Trustworthiness and publishing. *Eur J Gen Pract* 2018;24:120–4. doi:10.1080/13814788.2017.1375092
17 Butler-Kisber L. *Qualitative Inquiry: Narrative and Arts-Informed Perspectives*. 1st ed. Los Angeles: : SAGE 2010.
18 Nicholls D. Qualitative research. Part 3: Methods. *Int J Ther Rehabil* 2017;24:114–21. doi:10.12968/ijtr.2017.24.3.114
19 Lincoln YS, Guba EG. *Naturalistic Inquiry*. 1st ed. California: : SAGE 1985.
20 Birt L, Scott S, Cavers D, *et al.* Member Checking: A Tool to Enhance Trustworthiness or Merely a Nod to Validation? *Qual Health Res* 2016;26:1802–11. doi:10.1177/1049732316654870
21 World Bank. World Bank List of Economies 2019. 2019.https://datahelpdesk.worldbank.org/knowledgebase/articles/906519-world-bank-country-and-lending-groups (accessed 21 Nov 2019).
22 Moser A, Korstjens I. Series: Practical guidance to qualitative research. Part 3: Sampling, data collection and analysis. *Eur J Gen Pract* 2018;24:9–18. doi:10.1080/13814788.2017.1375091
23 Polit D, Beck C. *Resource Manual for Nursing Research: Generating and Assessing Evidence for Nursing Practice*. 9th ed. Lippincott Williams & Wilkins 2011.

Author Contributions

The concept of this study was conceived by AGK and PJH as part of Theme 3D of the NIHR Global Health Research, with further study design by CW, AK and BS. CW wrote the first draft of the protocol, with BS preparing the protocol for publication. TB, INE, CK, MK, DJFS, AMR and AF suggested amendments to the protocol and edited the final manuscript.

Funding Statement

This research was supported by National Institute for Health Research (NIHR) Global Health Research Group on Neurotrauma (grant number 16/137/105) using UK aid from the UK Government.

Competing Interests Statement

Angelos Kolias and Peter Hutchinson are supported by the National Institute for Health Research (NIHR) Cambridge Biomedical Research Centre and the NIHR Global Health Research Group on Neurotrauma. Peter Hutchinson is also supported by a NIHR Research Professorship. The NIHR Global Health Research Group on Neurotrauma was commissioned by the United Kingdom NIHR using Official Development Assistance funding (project no. 16/137/105). The views expressed in this manuscript are those of the authors and are not necessarily those of the United Kingdom National Health Service, NIHR, or the Department of Health. Drs Esene, Karekezi, Khan, Solla and Kolias are members of the Young Neurosurgeons committee of the World Federation of Neurosurgical Societies. The committee is supporting this project.

For peer review only

BMJ Open

Neurotrauma physicians’ perspectives on the contextual challenges associated with Long-term follow-up following Traumatic Brain Injury in Low- and Middle-income countries: a qualitative study protocol

Journal:	*BMJ Open*
Manuscript ID	bmjopen-2020-041442.R1
Article Type:	Protocol
Date Submitted by the Author:	12-Dec-2020
Complete List of Authors:	Smith, Brandon; Addenbrooke's Hospital, Department of Clinical Neurosciences, Division of Neurosurgery; University of Cambridge, NIHR Global Health Research Group on Neurotrauma<br>Whiffin, Charlotte; University of Derby, College of Health of Social Care; University of Cambridge, NIHR Global Health Research Group on Neurotrauma<br>Esene, Ignatius ; University of Bamenda, Neurosurgery Division, Faculty of Health Sciences<br>Karekezi, Claire; Rwanda Military Hospital, Department of Neurosurgery<br>Bashford, Tom; University of Cambridge, NIHR Global Health Research Group on Neurotrauma<br>Mukhtar Khan, Muhammad; Northwest General Hospital & Research Center, Neurosurgery; University of Cambridge, NIHR Global Health Research Group on Neurotrauma<br>Fontoura Solla, Davi; University of São Paulo Department of Neurosciences and Behaviour Sciences; University of Cambridge, NIHR Global Health Research Group on Neurotrauma<br>Indira Devi, Bhagavatula; National Institute of Mental Health and Neuro Sciences; University of Cambridge, NIHR Global Health Research Group on Neurotrauma<br>Hutchinson, Peter; Addenbrooke's Hospital, Department of Clinical Neurosciences, Division of Neurosurgery; University of Cambridge, NIHR Global Health Research Group on Neurotrauma<br>Kolias, Angelos; Addenbrooke's Hospital, Department of Clinical Neurosciences, Division of Neurosurgery; University of Cambridge, NIHR Global Health Research Group on Neurotrauma<br>Figaji, Anthony; University of Cape Town; University of Cambridge, NIHR Global Health Research Group on Neurotrauma<br>Rubiano, Andres M; Universidad El Bosque, Neurosciences Institute, Department of Neurosurgery; University of Cambridge, NIHR Global Health Research Group on Neurotrauma
**Primary Subject Heading**:	Neurology
Secondary Subject Heading:	Global health, Surgery, Qualitative research
Keywords:	QUALITATIVE RESEARCH, NEUROSURGERY, Neurological injury <

	NEUROLOGY

I, the Submitting Author has the right to grant and does grant on behalf of all authors of the Work (as defined in the below author licence), an exclusive licence and/or a non-exclusive licence for contributions from authors who are: i) UK Crown employees; ii) where BMJ has agreed a CC-BY licence shall apply, and/or iii) in accordance with the terms applicable for US Federal Government officers or employees acting as part of their official duties; on a worldwide, perpetual, irrevocable, royalty-free basis to BMJ Publishing Group Ltd ("BMJ") its licensees and where the relevant Journal is co-owned by BMJ to the co-owners of the Journal, to publish the Work in this journal and any other BMJ products and to exploit all rights, as set out in our licence.

The Submitting Author accepts and understands that any supply made under these terms is made by BMJ to the Submitting Author unless you are acting as an employee on behalf of your employer or a postgraduate student of an affiliated institution which is paying any applicable article publishing charge ("APC") for Open Access articles. Where the Submitting Author wishes to make the Work available on an Open Access basis (and intends to pay the relevant APC), the terms of reuse of such Open Access shall be governed by a Creative Commons licence – details of these licences and which Creative Commons licence will apply to this Work are set out in our licence referred to above.

Other than as permitted in any relevant BMJ Author's Self Archiving Policies, I confirm this Work has not been accepted for publication elsewhere, is not being considered for publication elsewhere and does not duplicate material already published. I confirm all authors consent to publication of this Work and authorise the granting of this licence.

For peer review only

Title page

Full title: Neurotrauma physicians' perspectives on the contextual challenges associated with Long-term follow-up following Traumatic Brain Injury in Low- and Middle-income countries: a qualitative study protocol
Short title: Long-term follow-up of TBI in LMICs

Brandon G Smith* [1 2] , Charlotte J Whiffin* [1 2 3], Ignatius N Esene[4], Claire Karekezi[5], Tom Bashford[2], Muhammad Mukhtar Khan[2 6], Davi J Fountoura Solla[2 7], Bhagavatula Indira Devi[2 8], Peter J Hutchinson[1 2], Angelos G Kolias[1 2], Anthony Figaji[2 9], Andres M Rubiano[2 10]

* joint first authors

[1] Division of Neurosurgery, Department of Clinical Neurosciences, Addenbrooke's Hospital and University of Cambridge, Cambridge, U.K.
[2] NIHR Global Health Research Group on Neurotrauma, University of Cambridge, Cambridge, UK
[3] College of Health and Social Care, University of Derby, U.K.
[4] Neurosurgery Division, Faculty of Health Sciences, University of Bamenda, Cameroon
[5] Rwanda Military Hospital, Department of Neurosurgery, Rwanda
[6] Northwest School of Medicine & Northwest general Hospital & Research Centre, Peshawar, Pakistan
[7] University of São Paulo, Brazil
[8] Department of Neurosurgery, NIMHANS, Bangalore, India
[9] University of Cape Town, Cape Town, South Africa
[10] Institute of Neurosciences and Neurosurgery, El Bosque University, Bogotá, Colombia

Corresponding author
Angelos G. Kolias
Clinical Lecturer in Neurosurgery
Division of Neurosurgery
Box 167
University of Cambridge
Cambridge Biomedical Campus
Cambridge, CB2 0QQ,
United Kingdom

Email address: ak721@cam.ac.uk

For peer review only

ABSTRACT

Introduction

Traumatic Brain Injury is a global public health concern; however, low- and middle-income countries (LMICs) face the greatest burden. The World Health Organisation recognise the significant differences between patient outcomes following injuries in high-income countries versus those in LMICs. Outcome data is not reliably recorded in LMICs and despite improved injury surveillance data, data on disability and long-term functional outcomes remains poorly recorded. Therefore, the full picture of outcome post-TBI in LMICs is largely unknown.

Methods and Analysis

This is a cross-sectional pragmatic qualitative study using individual semi-structured interviews with physicians who have experience of neurotrauma in LMICs. The aim of this study is to understand the contextual challenges associated with long-term follow-up of patients following traumatic brain injury in LMICs. For the purpose of the study, we define 'Long-term' as any data collected following discharge from hospital. We aim to conduct individual semi-structured interviews with 24 to 48 neurosurgeons. Interviews will be recorded and transcribed verbatim. A reflexive thematic analysis will be conducted supported by NVIVO software.

Ethics and Dissemination

The University of Cambridge Psychology Research Ethics Committee approved this study in February 2020. Ethical issues within this study include consent, confidentiality and anonymity, and data protection. Participants will provide informed consent and their contributions will be kept confidential. Participants will be free to withdraw at any time without penalty; however, their interview data can only be withdrawn up to one week after data collection. Findings generated from the study will be shared with relevant stakeholders such as the World Federation of Neurosurgical Societies and disseminated in conference presentations and journal publications.

Registration*: Not applicable.*

ARTICLE SUMMARY

Study Strengths and Limitations

- This study is among the first to examine the contextual challenges associated with long-term follow-up of patients following traumatic brain injury in LMICs.
- Findings generated from this study will enable us to establish an understanding of the ways long-term follow-up of neurotrauma outcomes can be successfully recorded in LMICs, and may facilitate the ongoing development of cost effective, novel follow-up technologies.
- Through our non-probability sampling technique and necessity for a small sample size, not all LMICs will be represented in this study, however an economic and geographic spread is sought.
- Due to the resources available and the need to conduct in-depth interviews and subsequent qualitative analysis, it was not possible to recruit non-English speaking physicians.

Key Words:

- Traumatic Brain Injury
- Qualitative Research
- Global Neurosurgery
- Follow-up

Word count: 3760

For peer review only

INTRODUCTION

Background

Traumatic Brain Injury (TBI) is a global public health concern; however, low- and middle-income countries (LMICs) face the greatest burden, where 85% of the world's population live[1,2]. Each year, an estimated 69 million cases of TBI are reported globally[3]. The World Health Organisation recognise the significant differences between patient outcomes following injuries in high-income countries (HICs) versus those in LMICs[4], and that almost 90% of deaths occurring from injuries occur in LMICs[5,6]. In high-income countries improvements in surgical interventions and medical management has meant rapid increases in survival rates and improved outcomes. In LMICs fewer resources, different treatment practices and reduced availability of surgical interventions has meant these countries have not all seen the same improvements in outcome post-TBI[7]. Despite a lack of robust data in the literature base to accurately be able to describe the incidence and mortality rates from TBI in LMICs, proportionally, LMICs experience three times as many cases of TBI when compared to HICs[3].

Acknowledging heterogeneity within both LMICs and HICs, as a whole outcome data is generally not reliably recorded in LMICs, and despite improved injury surveillance data, data on disability and long-term functional outcomes remains poorly recorded[8]. Therefore, the full picture of outcome post-TBI in LMICs is largely unknown. Laytin et al (2018) described the difficulty of collecting outcome data in resource poor settings, specifically Sub-Saharan Africa. Collection of outcome data in these settings was typically conducted within the emergency department or at hospital discharge rather than at points after discharge. Challenges of collecting outcome data included weak healthcare infrastructure and limited regular follow-up of trauma patients[8].

Despite such challenges the collection of long-term outcomes is essential, as it provides epidemiological data to properly determine the full burden of injury and assess the efficacy of patient treatment and management decisions in LMICs[8,9] In addition Mock et al.,[4] argues that to fully understand the full burden of disease, disability must be evaluated as well as death. The collection of long-term outcomes is additionally important in the implementation and continuation of registries. These resources serve to provide opportunities for continuous quality improvement and enable trials of interventions in these populations – the results of these registries can themselves be incorporated into legislation and specific care pathways[10]. Therefore, ways must be found to collect more complex long-term outcomes following TBI in LMICs that are directly comparable to the outcome data being collected in most high-income countries. Further adding to this challenge is a lack of clarity in the global definition of 'long-term' in neurotrauma, and what period this includes; a study of long-term outcomes after paediatric head trauma in Uganda defined long-term as anything 1-2 years post-injury[11], whilst studies from France and Sweden suggest long-term as periods spanning at least 3 years and beyond[12,13].

In turn, we must be mindful of the complex relationship between the economic status of each country and the quality of their neurosurgical follow-up provision. Many LMICs have highly heterogeneous health systems, and countries with a low gross national income per capita may still support well-functioning neurosurgical centres offering a high level of follow-up care. Although, on average, poorer countries might be expected to demonstrate worse follow-up as a function of resource limitation, we must be careful not to assume an association at the hospital level.

Rationale

This study is the first in a three-phase project which aims to explore long-term follow-up in LMICs, its challenges, facilitators and technological solutions to augment current practices. The existing literature appears to support an association between the utilisation of trauma registries, quality improvement programmes, and improved long-term outcomes. This association has been used to suggest a role for systems science approaches to improving neurotrauma care, particularly in LMICs[6,10,14]. A preliminary review of the evidence base revealed how little empirical literature had been published about the difficulties long-term follow-up poses within LMICs, and what specific contextual challenges exist within these countries. Without an in-depth understanding of these contextual challenges it was not possible to begin work on technological solutions to improve the collection of long-term outcomes in these countries. Therefore, an exploratory qualitative study was designed to provide an in-depth understanding of neurotrauma physicians' attitudes, experiences, and perspectives of follow-up. This foundation of understanding will make a meaningful contribution to the limited empirical evidence base while also informing later phases of the project examining technological solutions in low resources settings using quantitative methods.

For peer review only - http://bmjopen.bmj.com/site/about/guidelines.xhtml

This project is part of the NIHR Global Health Group on Neurotrauma and contributes to its strategic aims. The NIHR Global Health Group on Neurotrauma commenced in 2017 aiming to understand current systems and challenges in TBI clinical care within LMICs and as part of this acknowledged that the collection of long-term outcomes needed to be improved. Although beyond the scope of this particular project, results of this study may warrant an investigation into how well long-term follow-up is conducted across the spectrum of high-income country neurosurgical centres, that are not directly conducting or engaging in long-term outcome studies for objective comparison. In addition, we will be careful to ensure our results are not compared to presumptions, or to centres with follow-up efforts motivated only by engagement in international studies.

Theoretical Framework

This study uses a qualitative methodology which is especially useful to explore complex phenomena[15] when little is known about a topic or where new insights are required. Qualitative inquiry is committed to subjectivity and the collection of rich in-depth data through the examination of participant's viewpoints[15]. Pragmatic qualitative research, also known as descriptive and generic qualitative research, does not align itself to any specific philosophical perspective and therefore allows flexibility of methods that enables the study to be designed in an appropriate way to address the research question[16]. Pragmatic research lies within the naturalistic paradigm which recognises the existence of multiple realities and studies real-world situations by having little impact on the phenomena under investigation[17]. Pragmatic qualitative studies aim to achieve in-depth understanding prioritising literal description first and then understanding phenomena in a deeper sense through analysis and interpretation of how people draw meaning from their experiences[18]. The goal of such research is to provide an account of the "experiences, events and process that most people (researchers and participants) would agree are accurate"[19] [p128]. In addition, Sullivan-Bolyai, et al.[19] state qualitative descriptive designs are particularly useful when researching populations of other cultures and as such would be well suited to the aims of this study.

Research Aim

The aim of this study is to understand the contextual challenges associated with long-term follow-up of patients following traumatic brain injury in LMICs. For the purpose of the study we define 'Long-term' as any data collected following discharge from hospital.

Objectives

1. To determine current approaches to long-term follow up of patients following TBI in LMICs
2. To explore the challenges associated with long-term follow-up of patients following TBI in LMICs.
3. To collaboratively offer recommendations for appropriate solutions that will facilitate long-term follow-up of patients following TBI in LMICs based on findings and themes identified

METHODS AND ANALYSIS

Study Design

We propose a cross-sectional pragmatic qualitative study using individual semi-structured interviews with physicians who have experience of neurotrauma in LMICs. Despite the pragmatic nature of this study, the naturalistic paradigm in which it is situated aims to interpret individual experiences and employs a subjective epistemology which asserts universal knowledge is not possible[20]. A purposive sampling strategy will be employed to recruit 24 - 48 participants from LMIC countries participating in the NIHR GHRGN. Methods include semi-structured telephone/online video interviews and the collection of relevant demographic and professional data.

Data collection

This study will collect data through semi-structured telephone or online video interviews, conducted by a single interviewer (BS). Semi-structured interviews are the cornerstone of qualitative methodology as they facilitate rich data gathering and in-depth exploration of the phenomenon under investigation. As such semi-structured interviews were the natural choice for data collection in this study. Telephone and online video methods were chosen over face-to-face for pragmatic reasons given the geographic location of the participants and the interviewer. We will also collect some limited demographic data that will help us analyse and interpret the similarities and differences between the data sets (See Box One).

For peer review only - http://bmjopen.bmj.com/site/about/guidelines.xhtml

Specific Demographic data will be collected using an electronic form (see Box One).

Age<br>Sex<br>Country of residence<br>Occupation<br>Years of experience<br>Years working in neurotrauma<br>Practice setting
Box One: Demographic Data

Details of the interview questions are presented in Box Two, and participants will be made aware of these prior to the interview so they have the opportunity to consider their response. Questions are open ended to encourage a rich dialogue and participants will be free to elaborate on their answers adding additional detail as required. To encourage more in-depth responses prompts, probes and follow-up questions will be used. In this sense the questions in Box Two will be used more as a guide than an explicit framework, which is consistent with a semi-structured approach. It is anticipated that the interviews will last up to 60 minutes and will be conducted by BS following a pilot of the interview guide. Interviews will be recorded using a digital recorder.

Current follow-up

A. Please can you tell me about how and when your patients are currently followed-up following discharge from a TBI?
   a. What outcomes are measured?
   b. What needs are assessed?
   c. What referrals are made?
   d. What type of contact system do you have in place (face-to-face, telephone, telemedicine)?

Definition and understanding of long-term outcomes

A. What do you understand by the use of ‘long- term’ in the context of outcomes post-TBI?
B. Do you think your definition and understanding of ‘long-term outcomes’ in your country are the same as those in other countries?
C. Do you know or use any type(s) of outcome classification for follow-up?

Attitudes towards long-term follow-up

A. What do you think about the need for long-term follow-up of patients following TBI in your setting?
B. What would you consider to be the main benefits of long-term follow-up of TBI patients?
C. What do you consider to be the challenges associated with long-term follow-up of your patients with TBI?
D. Are these challenges more related to: health system aspects, national, regional, or local administrative aspects, institutional aspects, resources for care aspects?
E. Are there any other issues that need to be considered for long-term follow-up in your own setting and wider country?

Long-term follow-up in LMIC

A. Thinking more broadly now about other Low- and Middle-Income Countries, do you have any other thoughts about long-term follow-up in these other LMICs
B. What else might be required in LMICs to facilitate long-term follow-up of patients following TBI?

Possible interventions to facilitate follow-up in LMIC

A. Do you know what types of technology patients and families have access to that could help in follow-up of patients following TBI?
B. What kind of technology would help in your country to record long-term follow-up?
C. Are you aware of any specific mobile health, telemedicine, or phone interview options for long-term follow-up in your institution/state/country?
D. Does your service or institution participate in a trauma or neurotrauma quality improvement program including the use of clinical registries with outcome measures?

Box Two: Interview guide

For peer review only

Study Setting

Participants will be recruited from any country identified as low or middle income as defined by the 2017-2018 World Bank list of economies[21]. Access to participants will be negotiated using the research group's project lead and collaborators from the twelve countries in the NIHR Global Health Research Group on Neurotrauma listed in Box Three. New collaborators in the group, including the Philippines and Zimbabwe, in addition to participants from institutions from LMICs not yet part of the group, will be invited to participate as necessary in order to attain the defined sample size.

1. BRAZIL<br>2. COLOMBIA<br>3. ETHIOPIA<br>4. INDIA<br>5. INDONESIA<br>6. MALAYSIA	7. MYANMAR<br>8. NIGERIA<br>9. PAKISTAN<br>10. SOUTH AFRICA<br>11. TANZANIA<br>12. ZAMBIA
Box Three: LMICs in the NIHR Global Health Research Group on Neurotrauma	

Eligibility Criteria

To be eligible for this study the person must be a practising physician within an LMIC and have experience of managing neurotrauma, so they have experience of treatment and discharge of patients with TBI. We are requesting the physicians all have at least two years' experience of neurotrauma and also some experience of research, so they have more insight into the complexities of long-term follow-up of neurotrauma patients and understand the research process. Due to availability of resources participants will have to be fluent in English. Experience of working with collaborators would suggest this criterion will not limit participation in the study. All participants will have to provide informed consent by agreeing to the criteria within the consent form.

Inclusion criteria

• Physician within a LMIC with experience of managing neurotrauma<br>• At least two years' experience of neurotrauma<br>• Experience of collecting, or attempting to collect, TBI outcome data<br>• Self-declared fluency in spoken English<br>• Able to provide informed consent
Box Four: Inclusion criteria

Sample

The main criticism of qualitative studies stems from the necessity for small samples. Smaller samples facilitate depth of inquiry and rich interpretation. However, according to Sullivan-Bolyai, et al.[19] qualitative descriptive studies can accommodate more moderate sample sizes than theoretically informed studies because there is no requirement to generate theory. Therefore Sullivan-Bolyai, et al.[19] recommends a sample size of 20-50 participants.

Moser and Korstjens[22] explain that a guiding principle of sampling in qualitative studies is to sample up until data saturation is reached. Data saturation is a qualitative principle which is a point whereby the researchers are confident that new data will not reveal new information[23]. However, data saturation is a contentious issue in reflexive TA which advocates a less prescriptive approach to sampling and asks the researcher to make an interpretive judgement about when to stop[24].

In line with this view, sample size in this study was further informed by a desire to explore a variety of views from physicians within a range of LMICs. A non-probability, purposive sample is therefore required. We will aim to have representation from a range of countries and, if possible, a range of regions within these countries. Therefore, we propose a sample size of 24 - 48 participants and will comment further on principles of data saturation in the final paper.

For peer review only - http://bmjopen.bmj.com/site/about/guidelines.xhtml

Recruitment

We will begin by seeking to recruit 2-3 neurotrauma physicians from the collaborating countries in the NIHR Global Health Research Group on Neurotrauma, consisting of 12 LMICs (Box Three), with new collaborators from Zimbabwe and the Philippines. We will also be placing a call through social media (Twitter and WhatsApp) to maximise the opportunity for recruitment from any physicians not subscribed to the GHRGN mailing list or from other LMICs not listed as collaborating regions.

The local PIs will be written to through the group's previously established mailing lists, with information pertaining to the study including the aims of the study, inclusion criteria (Box Four) for participants and data collection methods. We will then ask the local PIs to forward information about the study to relevant physicians (NIHR GHRGN collaborators are also eligible to participate). For social media recruitment an infographic was developed for the study and distributed through Twitter as an open call for participants. Those who wished to express an interest can contact the lead author (BS) for more information. Information forwarded to participants will include a letter of invitation, participant information sheet, and contact details for the research team. If interested in participating an initial telephone call will be arranged to review the participant information sheet and the requirements of the study. If the physician still wishes to continue, we will schedule a telephone interview. We will leave a minimum of one week in-between this initial contact and arrangement of the interview to allow a cooling off period. If an insufficient number of people have responded to the first call for participants a second request for participation will be made two – three weeks later. Data will be collected over a 12-month period, beginning March 2020.

It is essential that participants join this study of their own free will. However, the notion that junior colleagues may have a 'reciprocal obligation' to assist their more senior colleagues, and the institution, in participating in global research is appreciated. To safeguard against potential coercion, we will highlight to our participants that they are under no obligation to take part. We will discuss this specifically during the pre-consent meeting and ensure they understand that no-one will be informed if they chose, or do not chose, to participate in the study. Participants will not be provided with any financial reimbursement, as no out of pocket expenses are anticipated.

Data Analysis

Analysis within pragmatic qualitative studies should stay close to the text, and does not require "highly abstract rendering of data"[18]. Staying close to the text means early coding decisions rely heavily on what participants actually said with interpretation applied more substantially in later phases of analysis. Studies which are independent of theory, can and often do use thematic analysis[15,25]. According to Braun and Clarke[25] thematic analysis can reveal a rich, detailed and complex exploration of the data and despite its 'descriptive' title will end with some necessary interpretation of the data[18] through discovery of shared and varied patterns of understanding[26]. Indeed Vaismoradi, et al.[15] contends that thematic approaches actually attends little to the description of the data set and instead requires interpretation of the various aspects of the phenomena under investigation. It is therefore proposed that a six stage Braun and Clarke[25] thematic analysis will be conducted (See Box Five). Congruent with Braun and Clarke's more recent commentary on thematic analysis, this study aligns itself to an approach described as 'reflexive TA'[27].

To complete the analysis audio files from interviews will first be transcribed verbatim by a transcription service and checked for accuracy by BS. Codes will be assigned to individual transcripts and will be supported by the use of NVIVO software, allowing researchers to organise the data, share coding decisions and confirm the origins of interpretation. Similar codes will be grouped together, and initial themes identified. Themes will then be reviewed, revised and final themes named and agreed upon within the research team. BS will take lead on the data analysis, supported by CW in the initial coding stages. This process of coding and theme building is not about reaching consensus between coders about the way codes were applied or their interpretation[27]. Instead, this is a collaborative process to advance understanding of the data and develop a more nuanced and reflexive interpretation congruent with our epistemological position. We will then report our final interpretive themes and the supporting sub-themes identified in the analysis. These will be defended through critical discussion and the origins of the themes evidenced through the presentation of anonymised direct quotes. A more in-depth discussion of the data analysis process and audit trail will be presented in the findings paper.

BS is a PhD student and fourth year medical student who has undertaken training in qualitative methods and analysis. CW is an experienced qualitative researcher and nurse academic.

For peer review only - http://bmjopen.bmj.com/site/about/guidelines.xhtml

1. Familiarising yourself with your data<br>2. Generate initial codes<br>3. Searching for themes<br>4. Reviewing themes<br>5. Defining and naming themes<br>6. Produce the report
Box Five: Braun and Clarke [25] thematic analysis framework

Rigour

Validity, generalizability and reliability have little relevance in qualitative research[28,29]. Instead qualitative research is judged by more appropriate criteria such as credibility and trustworthiness[30]. These hallmarks of quality determine the rigour of a qualitative study. In this study we intend to include a number of strategies advocated by Nicholls[30] to increase the rigour of the methods and consequently the final findings. First, following a reflexive, interpretive approach our findings will be strengthened through the critical dialogue between members of the research team, principally BS and CW, and subsequently with our co-authors. In addition, we propose using member checking as advocated by Lincoln and Guba[31] as crucial to establishing credibility in qualitative studies and according to Birt, et al.[32] can overcome issues of researcher bias and increases transferability of findings. In this regard, we will first return any transcripts where transcription was not possible due to the quality of recording to ask for clarification of specific sections of the interview if necessary. Second, we will share initial themes identified from the data with participants so they can add further insight to the interpretation of findings if they wish. These contributions will be added to the final analysis of data.

ETHICS & DISSEMINATION

This is an open, online study with global participants, with the primary investigator and principal research team all based in the UK. Although including overseas respondents, as this research is hosted and delivered by the UK, favourable opinion is only sought from a UK ethics committee.

Subsequently, in February 2020 The University of Cambridge Psychology Research Ethics Committee reviewed this study and provided favourable ethical opinion (Ref: PRE.2020.010), and thus is in line with the Concordat to Support Research Integrity from Universities UK, Research Councils UK and the UK Research Integrity Office (2013), International Ethical Guidelines for Health-related Research Involving Humans, Council for International Organizations of Medical Sciences (CIOMS) 2016, The World Medical Association's (WMA) Declaration of Helsinki and Good Clinical Practice (the ICH GCP R2 2016) as applicable. The University of Cambridge is the sponsor and appropriate insurance is in place. Ethical issues within this study include consent, confidentiality and anonymity, and data protection.

All participants will be required to provide informed consent after a cooling-off period of at least a week after the participant information sheet has been delivered. Participants can withdraw at any time without penalty, with a grace period of a week given after completion of the interview to withdraw their interview data, after which withdrawal of data will not be possible as analysis will have commenced.

All information will be kept strictly confidential, with all information generated to be stored on a GDPR compliant confidential server. A unique study ID will be allocated to all participants and used to anonymise personal data. Audio files will be immediately transferred to an encrypted folder and the data on the recording device destroyed, interview data will be anonymised upon transcription and other personal information changed or removed once checked by the research team. Participants will be told that anonymised quotes will be published in the findings of this study; however, despite this anonymity it is possible that their contributions may be recognisable to others reading the study. We will also be seeking consent to disclose region and level of income associated with their country of origin against participant quotes used in the publication of findings, as this will be important to the contextual understanding of the study.

For peer review only

Patient & Public Involvement

This study seeks to ascertain the views of physicians, and thus patient and public involvement was not considered to be as relevant in this context. However, we recognise the value of working with key stakeholders to develop research and therefore asked for peer review of this study by collaborating members of the GHRGN. Their comments informed the final study design.

Dissemination

When we have completed the study, we will produce a study summary which we will offer a copy to participants via email. Study findings will also be disseminated internationally through the GHRGN network and other relevant stakeholders such as the World Federation of Neurosurgical Societies, in addition to appropriate conferences, peer reviewed journal publications and social media.

Study Limitations

Understandably, through the non-probability approach to sampling and the necessity for a small sample size, not all LMICs will be represented in this study, however an economic and geographic spread is sought. We anticipate developing considerable insight from our international cohort of participants with differing levels of income and localities, in addition to establishing an understanding of the ways long-term follow-up of neurotrauma outcomes can be successfully recorded in LMICs. Careful attention to nuances must be paid. We respect and have the foresight that there will be heterogeneity within LMICs as a whole, and even within countries themselves as to the current provision, capabilities and attitudes towards neurotrauma follow-up. We must be sensitive when categorising our findings across a spectrum based on the gross national income per capita. We acknowledge that some false dichotomy may exist in the dialogue regarding healthcare provision, particularly of the collection of long-term outcomes, and caution will be exercised in interpretation of our findings. Unfortunately, due to the resources available and the need to conduct in-depth interviews and subsequent qualitative analysis, it is not possible to recruit non-English speaking physicians.

Furthermore, we recognise that this study may present a narrow perspective on long-term follow-up by only presenting the views of neurotrauma physicians. Given the dearth of literature in this field this study will still make a valuable contribution to the evidence base. However, future studies are encouraged that are inclusive of professional groups such as nurses, allied health professionals and data managers/epidemiologists who may have a wider view on neurotrauma follow-up in LMICs.

Notwithstanding, to our knowledge this study is among the first to examine the contextual challenges associated with long-term follow-up of patients following traumatic brain injury in LMICs. Findings generated from this study will enable us to establish an understanding of the ways long-term follow-up of neurotrauma outcomes can be successfully recorded in LMICs. This knowledge may lend itself to facilitating the ongoing development of cost-effective and novel follow-up technologies in the future.

Author Contributions

The concept of this study was conceived by AGK and PJH as part of Theme 3D of the NIHR Global Health Research, with further study design by CW, AK and BS. CW wrote the first draft of the protocol, with BS preparing the protocol for publication. TB, INE, CK, MK, DJFS, AMR and AF suggested amendments to the protocol and edited the final manuscript.

Competing Interests Statement

Angelos Kolias and Peter Hutchinson are supported by the National Institute for Health Research (NIHR) Cambridge Biomedical Research Centre and the NIHR Global Health Research Group on Neurotrauma. Peter Hutchinson is also supported by a NIHR Research Professorship. The NIHR Global Health Research Group on Neurotrauma was commissioned by the United Kingdom NIHR using Official Development Assistance funding (project no. 16/137/105). The views expressed in this manuscript are those of the authors and are not necessarily those of the United Kingdom National Health Service, NIHR, or the Department of Health. Drs Esene, Karekezi, Khan, Solla and Kolias are members of the Young Neurosurgeons committee of the World Federation of Neurosurgical Societies. The committee is supporting this project.

Funding Statement

For peer review only - http://bmjopen.bmj.com/site/about/guidelines.xhtml

This research was supported by National Institute for Health Research (NIHR) Global Health Research Group on Neurotrauma (grant number 16/137/105) using UK aid from the UK Government.

References

1 Kolias AG, Rubiano AM, Figaji A, *et al.* Traumatic brain injury: global collaboration for a global challenge. *Lancet Neurol* 2019;18:136–7. doi:10.1016/S1474-4422(18)30494-0

2 De Silva MJ, Roberts I, Perel P, *et al.* Patient outcome after traumatic brain injury in high-, middle- and low-income countries: analysis of data on 8927 patients in 46 countries. *Int J Epidemiol* 2009;38:452–8. doi:10.1093/ije/dyn189

3 Dewan MC, Rattani A, Gupta S, *et al.* Estimating the global incidence of traumatic brain injury. *J Neurosurg* 2019;130:1080–97. doi:10.3171/2017.10.JNS17352

4 Mock C, Lormand J, Goosen J, *et al.* Guidelines for essential trauma care. Geneva, World Health Organization: 2004.

5 Norton R, Kobusingye O. Injuries. *N Engl J Med* 2013;368:1723–30. doi:10.1056/NEJMra1109343

6 Rubiano AM, Carney N, Chesnut R, *et al.* Global neurotrauma research challenges and opportunities. *Nature* 2015;527:S193–7. doi:10.1038/nature16035

7 Servadei F, Tropeano MP, Spaggiari R, *et al.* Footprint of Reports From Low- and Low- to Middle-Income Countries in the Neurosurgical Data: A Study From 2015 to 2017. *World Neurosurg* 2019;130:e822–30. doi:10.1016/j.wneu.2019.06.230

8 Laytin AD, Seyoum N, Azazh A, *et al.* Feasibility of telephone-administered interviews to evaluate long-term outcomes of trauma patients in urban Ethiopia. *Trauma Surg Acute Care Open* 2018;3:e000256. doi:10.1136/tsaco-2018-000256

9 Laytin AD, Debebe F. The burden of injury in low-income and middle-income countries: knowing what we know, recognising what we don't know. *Emerg Med J* 2019;:emermed-2019-208514. doi:10.1136/emermed-2019-208514

10 Mowafi H, Ngaruiya C, O'Reilly G, *et al.* Emergency care surveillance and emergency care registries in low-income and middle-income countries: conceptual challenges and future directions for research. *BMJ Glob Heal* 2019;4:e001442. doi:10.1136/bmjgh-2019-001442

11 Vaca SD, Xu LW, Nalwanga J, *et al.* Long-term follow-up of pediatric head trauma patients treated at Mulago National Referral Hospital in Uganda. *J Neurosurg Pediatr* 2018;23:125–32. doi:https://dx.doi.org/10.3171/2018.7.PEDS17601

12 Åhman S, Saveman B, Styrke J, *et al.* Long-term follow-up of patients with mild traumatic brain injury: A mixed-method study. *J Rehabil Med* 2013;45:758–64. doi:10.2340/16501977-1182

13 Ruet A, Bayen E, Jourdan C, *et al.* A Detailed Overview of Long-Term Outcomes in Severe Traumatic Brain Injury Eight Years Post-injury. *Front Neurol* 2019;10. doi:10.3389/fneur.2019.00120

14 LaGrone LN, Romaní Pozo DA, Figueroa JF, *et al.* Status of trauma quality improvement programs in the Andean region: What foundation do we have to build on. *Injury* 2017;48:1985–93. doi:10.1016/j.injury.2017.03.003

15 Vaismoradi M, Turunen H, Bondas T. Content analysis and thematic analysis: Implications for conducting a qualitative descriptive study. *Nurs Health Sci* 2013;15:398–405. doi:10.1111/nhs.12048

16 Smith J, Bekker H, Cheater F. Theoretical versus pragmatic design in qualitative research. *Nurse Res* 2011;18:39–51. doi:10.7748/nr2011.01.18.2.39.c8283

17 Bowen GA. Naturalistic inquiry and the saturation concept: a research note. *Qual Res* 2008;8:137–52. doi:10.1177/1468794107085301

18 Bradshaw C, Atkinson S, Doody O. Employing a Qualitative Description Approach in Health Care Research. *Glob Qual Nurs Res* 2017;4:233339361774228. doi:10.1177/2333393617742282

19 Sullivan-Bolyai S, Bova C, Harper D. Developing and refining interventions in persons with health disparities: The use of Qualitative Description. *Nurs Outlook* 2005;53:127–33. doi:10.1016/j.outlook.2005.03.005

20 Levers M-JD. Philosophical Paradigms, Grounded Theory, and Perspectives on Emergence. *SAGE Open* 2013;3:215824401351724. doi:10.1177/2158244013517243

21 World Bank. World Bank List of Economies 2019. 2019.https://datahelpdesk.worldbank.org/knowledgebase/articles/906519-world-bank-country-and-lending-groups (accessed 21 Nov 2019).

22 Moser A, Korstjens I. Series: Practical guidance to qualitative research. Part 3: Sampling, data collection and analysis. *Eur J Gen Pract* 2018;24:9–18. doi:10.1080/13814788.2017.1375091

23 Polit D, Beck C. *Resource Manual for Nursing Research: Generating and Assessing Evidence for Nursing Practice*. 9th ed. Lippincott Williams & Wilkins 2011.

For peer review only - http://bmjopen.bmj.com/site/about/guidelines.xhtml

24 Braun V, Clarke V. To saturate or not to saturate? Questioning data saturation as a useful concept for thematic analysis and sample-size rationales. *Qual Res Sport Exerc Heal* 2019;:1–16. doi:10.1080/2159676X.2019.1704846

25 Braun V, Clarke V. What can "thematic analysis" offer health and wellbeing researchers? *Int J Qual Stud Health Well-being* 2014;9:26152. doi:10.3402/qhw.v9.26152

26 Ulin PR, Robinson ET, Tolley EE, *et al. Qualitative Methods in Public Health: A Field Guide for Applied Research*. 2nd ed. 2016.

27 Braun V, Clarke V. Reflecting on reflexive thematic analysis. *Qual Res Sport Exerc Heal* 2019;11:589–97. doi:10.1080/2159676X.2019.1628806

28 Korstjens I, Moser A. Series: Practical guidance to qualitative research. Part 4: Trustworthiness and publishing. *Eur J Gen Pract* 2018;24:120–4. doi:10.1080/13814788.2017.1375092

29 Butler-Kisber L. *Qualitative Inquiry: Narrative and Arts-Informed Perspectives*. 1st ed. Los Angeles: : SAGE 2010.

30 Nicholls D. Qualitative research. Part 3: Methods. *Int J Ther Rehabil* 2017;24:114–21. doi:10.12968/ijtr.2017.24.3.114

31 Lincoln YS, Guba EG. *Naturalistic Inquiry*. 1st ed. California: : SAGE 1985.

32 Birt L, Scott S, Cavers D, *et al.* Member Checking: A Tool to Enhance Trustworthiness or Merely a Nod to Validation? *Qual Health Res* 2016;26:1802–11. doi:10.1177/1049732316654870

For peer review only

For peer review only - http://bmjopen.bmj.com/site/about/guidelines.xhtml

BMJ Open

Neurotrauma physicians’ perspectives on the contextual challenges associated with Long-term follow-up following Traumatic Brain Injury in Low- and Middle-income countries: a qualitative study protocol

Journal:	*BMJ Open*
Manuscript ID	bmjopen-2020-041442.R2
Article Type:	Protocol
Date Submitted by the Author:	21-Jan-2021
Complete List of Authors:	Smith, Brandon; Addenbrooke's Hospital, Department of Clinical Neurosciences, Division of Neurosurgery; University of Cambridge, NIHR Global Health Research Group on Neurotrauma<br>Whiffin, Charlotte; University of Derby, College of Health of Social Care; University of Cambridge, NIHR Global Health Research Group on Neurotrauma<br>Esene, Ignatius ; University of Bamenda, Neurosurgery Division, Faculty of Health Sciences<br>Karekezi, Claire; Rwanda Military Hospital, Department of Neurosurgery<br>Bashford, Tom; University of Cambridge, NIHR Global Health Research Group on Neurotrauma<br>Mukhtar Khan, Muhammad; Northwest General Hospital & Research Center, Neurosurgery; University of Cambridge, NIHR Global Health Research Group on Neurotrauma<br>Fontoura Solla, Davi; University of São Paulo Department of Neurosciences and Behaviour Sciences; University of Cambridge, NIHR Global Health Research Group on Neurotrauma<br>Indira Devi, Bhagavatula; National Institute of Mental Health and Neuro Sciences; University of Cambridge, NIHR Global Health Research Group on Neurotrauma<br>Hutchinson, Peter; Addenbrooke's Hospital, Department of Clinical Neurosciences, Division of Neurosurgery; University of Cambridge, NIHR Global Health Research Group on Neurotrauma<br>Kolias, Angelos; Addenbrooke's Hospital, Department of Clinical Neurosciences, Division of Neurosurgery; University of Cambridge, NIHR Global Health Research Group on Neurotrauma<br>Figaji, Anthony; University of Cape Town; University of Cambridge, NIHR Global Health Research Group on Neurotrauma<br>Rubiano, Andres M; Universidad El Bosque, Neurosciences Institute, Department of Neurosurgery; University of Cambridge, NIHR Global Health Research Group on Neurotrauma
**Primary Subject Heading**:	Neurology
Secondary Subject Heading:	Global health, Surgery, Qualitative research
Keywords:	QUALITATIVE RESEARCH, NEUROSURGERY, Neurological injury <

	NEUROLOGY

SCHOLARONE™
Manuscripts

I, the Submitting Author has the right to grant and does grant on behalf of all authors of the Work (as defined in the below author licence), an exclusive licence and/or a non-exclusive licence for contributions from authors who are: i) UK Crown employees; ii) where BMJ has agreed a CC-BY licence shall apply, and/or iii) in accordance with the terms applicable for US Federal Government officers or employees acting as part of their official duties; on a worldwide, perpetual, irrevocable, royalty-free basis to BMJ Publishing Group Ltd ("BMJ") its licensees and where the relevant Journal is co-owned by BMJ to the co-owners of the Journal, to publish the Work in this journal and any other BMJ products and to exploit all rights, as set out in our licence.

The Submitting Author accepts and understands that any supply made under these terms is made by BMJ to the Submitting Author unless you are acting as an employee on behalf of your employer or a postgraduate student of an affiliated institution which is paying any applicable article publishing charge ("APC") for Open Access articles. Where the Submitting Author wishes to make the Work available on an Open Access basis (and intends to pay the relevant APC), the terms of reuse of such Open Access shall be governed by a Creative Commons licence – details of these licences and which Creative Commons licence will apply to this Work are set out in our licence referred to above.

Other than as permitted in any relevant BMJ Author's Self Archiving Policies, I confirm this Work has not been accepted for publication elsewhere, is not being considered for publication elsewhere and does not duplicate material already published. I confirm all authors consent to publication of this Work and authorise the granting of this licence.

For peer review only

Title page

Full title: Neurotrauma physicians' perspectives on the contextual challenges associated with Long-term follow-up following Traumatic Brain Injury in Low- and Middle-income countries: a qualitative study protocol
Short title: Long-term follow-up of TBI in LMICs

Brandon G Smith* [1 2], Charlotte J Whiffin* [1 2 3], Ignatius N Esene[4], Claire Karekezi[5], Tom Bashford[2], Muhammad Mukhtar Khan[2 6], Davi J Fountoura Solla[2 7], Bhagavatula Indira Devi[2 8], Peter J Hutchinson[1 2], Angelos G Kolias[1 2], Anthony Figaji[2 9], Andres M Rubiano[2 10]

* joint first authors

[1] Division of Neurosurgery, Department of Clinical Neurosciences, Addenbrooke's Hospital and University of Cambridge, Cambridge, U.K.
[2] NIHR Global Health Research Group on Neurotrauma, University of Cambridge, Cambridge, UK
[3] College of Health and Social Care, University of Derby, U.K.
[4] Neurosurgery Division, Faculty of Health Sciences, University of Bamenda, Cameroon
[5] Rwanda Military Hospital, Department of Neurosurgery, Rwanda
[6] Northwest School of Medicine & Northwest general Hospital & Research Centre, Peshawar, Pakistan
[7] University of São Paulo, Brazil
[8] Department of Neurosurgery, NIMHANS, Bangalore, India
[9] University of Cape Town, Cape Town, South Africa
[10] Institute of Neurosciences and Neurosurgery, El Bosque University, Bogotá, Colombia

Corresponding author
Angelos G. Kolias
Clinical Lecturer in Neurosurgery
Division of Neurosurgery
Box 167
University of Cambridge
Cambridge Biomedical Campus
Cambridge, CB2 0QQ,
United Kingdom

Email address: ak721@cam.ac.uk

For peer review only

ABSTRACT

Introduction

Traumatic Brain Injury is a global public health concern; however, low- and middle-income countries (LMICs) face the greatest burden. The World Health Organisation recognise the significant differences between patient outcomes following injuries in high-income countries versus those in LMICs. Outcome data is not reliably recorded in LMICs and despite improved injury surveillance data, data on disability and long-term functional outcomes remains poorly recorded. Therefore, the full picture of outcome post-TBI in LMICs is largely unknown.

Methods and Analysis

This is a cross-sectional pragmatic qualitative study using individual semi-structured interviews with physicians who have experience of neurotrauma in LMICs. The aim of this study is to understand the contextual challenges associated with long-term follow-up of patients following traumatic brain injury in LMICs. For the purpose of the study, we define 'Long-term' as any data collected following discharge from hospital. We aim to conduct individual semi-structured interviews with 24 to 48 neurosurgeons, beginning February 2020. Interviews will be recorded and transcribed verbatim. A reflexive thematic analysis will be conducted supported by NVIVO software.

Ethics and Dissemination

The University of Cambridge Psychology Research Ethics Committee approved this study in February 2020. Ethical issues within this study include consent, confidentiality and anonymity, and data protection. Participants will provide informed consent and their contributions will be kept confidential. Participants will be free to withdraw at any time without penalty; however, their interview data can only be withdrawn up to one week after data collection. Findings generated from the study will be shared with relevant stakeholders such as the World Federation of Neurosurgical Societies and disseminated in conference presentations and journal publications.

Registration*: Not applicable.*

ARTICLE SUMMARY

Study Strengths and Limitations

- This study is among the first to examine the contextual challenges associated with long-term follow-up of patients following traumatic brain injury in LMICs.
- Findings generated from this study will enable us to establish an understanding of the ways long-term follow-up of neurotrauma outcomes can be successfully recorded in LMICs, and may facilitate the ongoing development of cost effective, novel follow-up technologies.
- Through our non-probability sampling technique and necessity for a small sample size, not all LMICs will be represented in this study, however an economic and geographic spread is sought.
- Due to the resources available and the need to conduct in-depth interviews and subsequent qualitative analysis, it was not possible to recruit non-English speaking physicians.

Key Words:

- Traumatic Brain Injury
- Qualitative Research
- Global Neurosurgery
- Follow-up

Word count: 3862

For peer review only

INTRODUCTION

Background

Traumatic Brain Injury (TBI) is a global public health concern; however, low- and middle-income countries (LMICs) face the greatest burden, where 85% of the world's population live[1,2]. Each year, an estimated 69 million cases of TBI are reported globally[3], with a substantial portion of these cases from falls and road traffic incidents [3,4]. In 2017, there were 521 million cases of all non-fatal injuries, a sharp increase from 354 million reported in 1990. When considering disability-adjusted life years (DALYs) from these injuries, road incidents and falls were found to be the main contributors. Haagsma et al. demonstrate that globally, age-standardised DALY rates increase with decreasing Socio-demographic Index (SDI) [5]. The World Health Organisation recognise the significant differences between patient outcomes following injuries in high-income countries (HICs) versus those in LMICs[6], and that almost 90% of deaths occurring from injuries occur in LMICs[7,8]. In high-income countries improvements in surgical interventions and medical management has meant rapid increases in survival rates and improved outcomes. In LMICs fewer resources, different treatment practices and reduced availability of surgical interventions has meant these countries have not all seen the same improvements in outcome post-TBI[9]. Despite a lack of robust data in the literature base to accurately be able to describe the incidence and mortality rates from TBI in LMICs, proportionally, LMICs experience three times as many cases of TBI when compared to HICs[3].

Acknowledging heterogeneity within both LMICs and HICs, as a whole outcome data is generally not reliably recorded in LMICs, and despite improved injury surveillance data, data on disability and long-term functional outcomes remains poorly recorded[10]. Therefore, the full picture of outcome post-TBI in LMICs is largely unknown. Laytin et al (2018) described the difficulty of collecting outcome data in resource poor settings, specifically Sub-Saharan Africa. Collection of outcome data in these settings was typically conducted within the emergency department or at hospital discharge rather than at points after discharge. Challenges of collecting outcome data included weak healthcare infrastructure and limited regular follow-up of trauma patients[10].

Despite such challenges the collection of long-term outcomes is essential, as it provides epidemiological data to properly determine the full burden of injury and assess the efficacy of patient treatment and management decisions in LMICs[10,11] In addition Mock et al.,[6] argues that to fully understand the full burden of disease, disability must be evaluated as well as death. The collection of long-term outcomes is additionally important in the implementation and continuation of registries. These resources serve to provide opportunities for continuous quality improvement and enable trials of interventions in these populations – the results of these registries can themselves be incorporated into legislation and specific care pathways[12]. Therefore, ways must be found to collect more complex long-term outcomes following TBI in LMICs that are directly comparable to the outcome data being collected in most high-income countries. Further adding to this challenge is a lack of clarity in the global definition of 'long-term' in neurotrauma, and what period this includes; a study of long-term outcomes after paediatric head trauma in Uganda defined long-term as anything 1-2 years post-injury[13], whilst studies from France and Sweden suggest long-term as periods spanning at least 3 years and beyond[14,15].

In turn, we must be mindful of the complex relationship between the economic status of each country and the quality of their neurosurgical follow-up provision. Many LMICs have highly heterogeneous health systems, and countries with a low gross national income per capita may still support well-functioning neurosurgical centres offering a high level of follow-up care. Although, on average, poorer countries might be expected to demonstrate worse follow-up as a function of resource limitation, we must be careful not to assume an association at the hospital level.

Rationale

This study is the first in a three-phase project which aims to explore long-term follow-up in LMICs, its challenges, facilitators and technological solutions to augment current practices. The existing literature appears to support an association between the utilisation of trauma registries, quality improvement programmes, and improved long-term outcomes. This association has been used to suggest a role for systems science approaches to improving neurotrauma care, particularly in LMICs[8,12,16]. A preliminary review of the evidence base revealed how little empirical literature had been published about the difficulties long-term follow-up poses within LMICs, and what specific contextual challenges exist within these countries. Without an in-depth understanding of these contextual challenges it was not possible to begin work on technological solutions to improve the

For peer review only - http://bmjopen.bmj.com/site/about/guidelines.xhtml

collection of long-term outcomes in these countries. Therefore, an exploratory qualitative study was designed to provide an in-depth understanding of neurotrauma physicians' attitudes, experiences, and perspectives of follow-up. For the purpose of this study a 'neurotrauma physician' is defined as any medical doctor or surgeon directly involved in the provision of acute neurotrauma care. This foundation of understanding will make a meaningful contribution to the limited empirical evidence base while also informing later phases of the project examining technological solutions in low resources settings using quantitative methods.

This project is part of the NIHR Global Health Group on Neurotrauma and contributes to its strategic aims. The NIHR Global Health Group on Neurotrauma commenced in 2017 aiming to understand current systems and challenges in TBI clinical care within LMICs and as part of this acknowledged that the collection of long-term outcomes needed to be improved. Although beyond the scope of this particular project, results of this study may warrant an investigation into how well long-term follow-up is conducted across the spectrum of high-income country neurosurgical centres, that are not directly conducting or engaging in long-term outcome studies for objective comparison. In addition, we will be careful to ensure our results are not compared to presumptions, or to centres with follow-up efforts motivated only by engagement in international studies.

Theoretical Framework

This study uses a qualitative methodology which is especially useful to explore complex phenomena[17] when little is known about a topic or where new insights are required. Qualitative inquiry is committed to subjectivity and the collection of rich in-depth data through the examination of participant's viewpoints[17]. Pragmatic qualitative research, also known as descriptive and generic qualitative research, does not align itself to any specific philosophical perspective and therefore allows flexibility of methods that enables the study to be designed in an appropriate way to address the research question[18]. Pragmatic research lies within the naturalistic paradigm which recognises the existence of multiple realities and studies real-world situations by having little impact on the phenomena under investigation[19]. Pragmatic qualitative studies aim to achieve in-depth understanding prioritising literal description first and then understanding phenomena in a deeper sense through analysis and interpretation of how people draw meaning from their experiences[20]. The goal of such research is to provide an account of the "experiences, events and process that most people (researchers and participants) would agree are accurate"[21] [p128]. In addition, Sullivan-Bolyai, et al.[21] state qualitative descriptive designs are particularly useful when researching populations of other cultures and as such would be well suited to the aims of this study.

Research Aim

The aim of this study is to understand the contextual challenges associated with long-term follow-up of patients following traumatic brain injury in LMICs. For the purpose of the study we define 'Long-term' as any data collected following discharge from hospital.

Objectives

1. To determine current approaches to long-term follow up of patients following TBI in LMICs
2. To explore the challenges associated with long-term follow-up of patients following TBI in LMICs.
3. To collaboratively offer recommendations for appropriate solutions that will facilitate long-term follow-up of patients following TBI in LMICs based on findings and themes identified

METHODS AND ANALYSIS

Study Design

We propose a cross-sectional pragmatic qualitative study using individual semi-structured interviews with physicians who have experience of neurotrauma in LMICs. Despite the pragmatic nature of this study, the naturalistic paradigm in which it is situated aims to interpret individual experiences and employs a subjective epistemology which asserts universal knowledge is not possible[22]. A purposive sampling strategy will be employed to recruit 24 - 48 participants from LMIC countries participating in the NIHR GHRGN. Methods include semi-structured telephone/online video interviews and the collection of relevant demographic and professional data.

Data collection

This study will collect data through semi-structured telephone or online video interviews, conducted by a single interviewer (BS). Semi-structured interviews are the cornerstone of qualitative methodology as they facilitate

For peer review only - http://bmjopen.bmj.com/site/about/guidelines.xhtml

rich data gathering and in-depth exploration of the phenomenon under investigation. As such semi-structured interviews were the natural choice for data collection in this study. Telephone and online video methods were chosen over face-to-face for pragmatic reasons given the geographic location of the participants and the interviewer. We will also collect some limited demographic data that will help us analyse and interpret the similarities and differences between the data sets (See Box One).

Specific Demographic data will be collected using an electronic form (see Box One).

Age<br>Sex<br>Country of residence<br>Occupation<br>Years of experience<br>Years working in neurotrauma<br>Practice setting
Box One: Demographic Data

Details of the interview questions are presented in Box Two, and participants will be made aware of these prior to the interview so they have the opportunity to consider their response. Questions are open ended to encourage a rich dialogue and participants will be free to elaborate on their answers adding additional detail as required. To encourage more in-depth responses prompts, probes and follow-up questions will be used. In this sense the questions in Box Two will be used more as a guide than an explicit framework, which is consistent with a semi-structured approach. It is anticipated that the interviews will last up to 60 minutes and will be conducted by BS following a pilot of the interview guide. Interviews will be recorded using a digital recorder.

Current follow-up

A. Please can you tell me about how and when your patients are currently followed-up following discharge from a TBI?
   a. What outcomes are measured?
   b. What needs are assessed?
   c. What referrals are made?
   d. What type of contact system do you have in place (face-to-face, telephone, telemedicine)?

Definition and understanding of long-term outcomes

A. What do you understand by the use of 'long- term' in the context of outcomes post-TBI?
B. Do you think your definition and understanding of 'long-term outcomes' in your country are the same as those in other countries?
C. Do you know or use any type(s) of outcome classification for follow-up?

Attitudes towards long-term follow-up

A. What do you think about the need for long-term follow-up of patients following TBI in your setting?
B. What would you consider to be the main benefits of long-term follow-up of TBI patients?
C. What do you consider to be the challenges associated with long-term follow-up of your patients with TBI?
D. Are these challenges more related to: health system aspects, national, regional, or local administrative aspects, institutional aspects, resources for care aspects?
E. Are there any other issues that need to be considered for long-term follow-up in your own setting and wider country?

Long-term follow-up in LMIC

A. Thinking more broadly now about other Low- and Middle-Income Countries, do you have any other thoughts about long-term follow-up in these other LMICs
B. What else might be required in LMICs to facilitate long-term follow-up of patients following TBI?

Possible interventions to facilitate follow-up in LMIC

A. Do you know what types of technology patients and families have access to that could help in follow-up of patients following TBI?
B. What kind of technology would help in your country to record long-term follow-up?
C. Are you aware of any specific mobile health, telemedicine, or phone interview options for long-term follow-up in your institution/state/country?

For peer review only - http://bmjopen.bmj.com/site/about/guidelines.xhtml

D. Does your service or institution participate in a trauma or neurotrauma quality improvement program including the use of clinical registries with outcome measures?	

Box Two: Interview guide

Study Setting

Participants will be recruited from any country identified as low or middle income as defined by the 2017-2018 World Bank list of economies[23]. Access to participants will be negotiated using the research group's project lead and collaborators from the twelve countries in the NIHR Global Health Research Group on Neurotrauma listed in Box Three. New collaborators in the group, including the Philippines and Zimbabwe, in addition to participants from institutions from LMICs not yet part of the group, will be invited to participate as necessary in order to attain the defined sample size.

1. BRAZIL	7. MYANMAR
2. COLOMBIA	8. NIGERIA
3. ETHIOPIA	9. PAKISTAN
4. INDIA	10. SOUTH AFRICA
5. INDONESIA	11. TANZANIA
6. MALAYSIA	12. ZAMBIA

Box Three: LMICs in the NIHR Global Health Research Group on Neurotrauma

Eligibility Criteria

To be eligible for this study the person must be a practising physician within an LMIC and have experience of managing neurotrauma, so they have experience of treatment and discharge of patients with TBI. We are requesting the physicians all have at least two years' experience of neurotrauma and also some experience of research, so they have more insight into the complexities of long-term follow-up of neurotrauma patients and understand the research process. Due to availability of resources participants will have to be fluent in English. Experience of working with collaborators would suggest this criterion will not limit participation in the study. All participants will have to provide written informed consent by agreeing to the criteria within the electronic consent form.

Inclusion criteria

- Physician within a LMIC with experience of managing neurotrauma
- At least two years' experience of neurotrauma
- Experience of collecting, or attempting to collect, TBI outcome data
- Self-declared fluency in spoken English
- Able to provide informed consent

Box Four: Inclusion criteria

Sample

The main criticism of qualitative studies stems from the necessity for small samples. Smaller samples facilitate depth of inquiry and rich interpretation. However, according to Sullivan-Bolyai, et al.[21] qualitative descriptive studies can accommodate more moderate sample sizes than theoretically informed studies because there is no requirement to generate theory. Therefore Sullivan-Bolyai, et al.[21] recommends a sample size of 20-50 participants.

Moser and Korstjens[24] explain that a guiding principle of sampling in qualitative studies is to sample up until data saturation is reached. Data saturation is a qualitative principle which is a point whereby the researchers are confident that new data will not reveal new information[25]. However, data saturation is a contentious issue in reflexive thematic analysis which advocates a less prescriptive approach to sampling and asks the researcher to make an interpretive judgement about when to stop[26].

For peer review only - http://bmjopen.bmj.com/site/about/guidelines.xhtml

In line with this view, sample size in this study was further informed by a desire to explore a variety of views from physicians within a range of LMICs. A non-probability, purposive sample is therefore required. We will aim to have representation from a range of countries and, if possible, a range of regions within these countries. Therefore, we propose a sample size of 24 - 48 participants and will comment further on principles of data saturation in the final paper.

Recruitment

We will begin by seeking to recruit 2-3 neurotrauma physicians from the collaborating countries in the NIHR Global Health Research Group on Neurotrauma, consisting of 12 LMICs (Box Three), with new collaborators from Zimbabwe and the Philippines. We will also be placing a call through social media (Twitter and WhatsApp) to maximise the opportunity for recruitment from any physicians not subscribed to the GHRGN mailing list or from other LMICs not listed as collaborating regions.

The local primary investigators (PIs) will be written to through the group's previously established mailing lists, with information pertaining to the study including the aims of the study, inclusion criteria (Box Four) for participants and data collection methods. We will then ask the local PIs to forward information about the study to relevant physicians (NIHR GHRGN collaborators are also eligible to participate). For social media recruitment an infographic was developed for the study and distributed through Twitter as an open call for participants. Those who wished to express an interest can contact the lead author (BS) for more information. Information forwarded to participants will include a letter of invitation, participant information sheet, and contact details for the research team. If interested in participating an initial telephone call will be arranged to review the participant information sheet and the requirements of the study. If the physician still wishes to continue, we will schedule a telephone interview. We will leave a minimum of one week in-between this initial contact and arrangement of the interview to allow a cooling off period. If an insufficient number of people have responded to the first call for participants a second request for participation will be made two – three weeks later. Data will be collected over a 12-month period, beginning March 2020.

It is essential that participants join this study of their own free will. However, the notion that junior colleagues may have a 'reciprocal obligation' to assist their more senior colleagues, and the institution, in participating in global research is appreciated. To safeguard against potential coercion, we will highlight to our participants that they are under no obligation to take part. We will discuss this specifically during the pre-consent meeting and ensure they understand that no-one will be informed if they chose, or do not chose, to participate in the study. Participants will not be provided with any financial reimbursement, as no out of pocket expenses are anticipated.

Data Analysis

Analysis within pragmatic qualitative studies should stay close to the text, and does not require "highly abstract rendering of data"[20]. Staying close to the text means early coding decisions rely heavily on what participants actually said with interpretation applied more substantially in later phases of analysis. Studies which are independent of theory, can and often do use thematic analysis[17,27]. According to Braun and Clarke[27] thematic analysis can reveal a rich, detailed and complex exploration of the data and despite its 'descriptive' title will end with some necessary interpretation of the data[20] through discovery of shared and varied patterns of understanding[28]. Indeed Vaismoradi, et al.[17] contends that thematic approaches actually attends little to the description of the data set and instead requires interpretation of the various aspects of the phenomena under investigation. It is therefore proposed that a six stage Braun and Clarke[27] thematic analysis will be conducted (See Box Five). Congruent with Braun and Clarke's more recent commentary on thematic analysis, this study aligns itself to an approach described as 'reflexive TA'[29].

To complete the analysis audio files from interviews will first be transcribed verbatim by a transcription service and checked for accuracy by BS. Codes will be assigned to individual transcripts and will be supported by the use of NVIVO software, allowing researchers to organise the data, share coding decisions and confirm the origins of interpretation. Similar codes will be grouped together, and initial themes identified. Themes will then be reviewed, revised and final themes named and agreed upon within the research team. BS will take lead on the data analysis, supported by CW in the initial coding stages. This process of coding and theme building is not about reaching consensus between coders about the way codes were applied or their interpretation[29]. Instead, this is a collaborative process to advance understanding of the data and develop a more nuanced and

For peer review only - http://bmjopen.bmj.com/site/about/guidelines.xhtml

reflexive interpretation congruent with our epistemological position. We will then report our final interpretive themes and the supporting sub-themes identified in the analysis. These will be defended through critical discussion and the origins of the themes evidenced through the presentation of anonymised direct quotes. A more in-depth discussion of the data analysis process and audit trail will be presented in the findings paper.

BS is a PhD student and fourth year medical student who has undertaken training in qualitative methods and analysis. CW is an experienced qualitative researcher and nurse academic.

1. Familiarising yourself with your data
2. Generate initial codes
3. Searching for themes
4. Reviewing themes
5. Defining and naming themes
6. Produce the report

Box Five: Braun and Clarke [27] thematic analysis framework

Rigour

Validity, generalizability and reliability have little relevance in qualitative research[30,31]. Instead qualitative research is judged by more appropriate criteria such as credibility and trustworthiness[32]. These hallmarks of quality determine the rigour of a qualitative study. In this study we intend to include a number of strategies advocated by Nicholls[32] to increase the rigour of the methods and consequently the final findings. First, following a reflexive, interpretive approach our findings will be strengthened through the critical dialogue between members of the research team, principally BS and CW, and subsequently with our co-authors. In addition, we propose using member checking as advocated by Lincoln and Guba[33] as crucial to establishing credibility in qualitative studies and according to Birt, et al.[34] can overcome issues of researcher bias and increases transferability of findings. In this regard, we will first return any transcripts where transcription was not possible due to the quality of recording to ask for clarification of specific sections of the interview if necessary. Second, we will share initial themes identified from the data with participants so they can add further insight to the interpretation of findings if they wish. These contributions will be added to the final analysis of data.

ETHICS & DISSEMINATION

This is an open, online study with global participants, with the primary investigator and principal research team all based in the UK. Although including overseas respondents, as this research is hosted and delivered by the UK, favourable opinion is only sought from a UK ethics committee.

Subsequently, in February 2020 The University of Cambridge Psychology Research Ethics Committee reviewed this study and provided favourable ethical opinion (Ref: PRE.2020.010), and thus is in line with the Concordat to Support Research Integrity from Universities UK, Research Councils UK and the UK Research Integrity Office (2013), International Ethical Guidelines for Health-related Research Involving Humans, Council for International Organizations of Medical Sciences (CIOMS) 2016, The World Medical Association's (WMA) Declaration of Helsinki and Good Clinical Practice (the ICH GCP R2 2016) as applicable. The University of Cambridge is the sponsor and appropriate insurance is in place. Ethical issues within this study include consent, confidentiality and anonymity, and data protection.

All participants will be required to provide written informed consent after a cooling-off period of at least a week after the participant information sheet has been delivered. Participants can withdraw at any time without penalty, with a grace period of a week given after completion of the interview to withdraw their interview data, after which withdrawal of data will not be possible as analysis will have commenced.

All information will be kept strictly confidential, with all information generated to be stored on a GDPR compliant confidential server. A unique study ID will be allocated to all participants and used to anonymise personal data. Audio files will be immediately transferred to an encrypted folder and the data on the recording device destroyed, interview data will be anonymised upon transcription and other personal information changed or

For peer review only - http://bmjopen.bmj.com/site/about/guidelines.xhtml

removed once checked by the research team. Participants will be told that anonymised quotes will be published in the findings of this study; however, despite this anonymity it is possible that their contributions may be recognisable to others reading the study. We will also be seeking consent to disclose region and level of income associated with their country of origin against participant quotes used in the publication of findings, as this will be important to the contextual understanding of the study.

Patient & Public Involvement

This study seeks to ascertain the views of physicians, and thus patient and public involvement was not considered to be as relevant in this context. However, we recognise the value of working with key stakeholders to develop research and therefore asked for peer review of this study by collaborating members of the GHRGN. Their comments informed the final study design.

Dissemination

When we have completed the study, we will produce a study summary which we will offer a copy to participants via email. Study findings will also be disseminated internationally through the GHRGN network and other relevant stakeholders such as the World Federation of Neurosurgical Societies, in addition to appropriate conferences, peer reviewed journal publications and social media.

Study Limitations

Understandably, through the non-probability approach to sampling and the necessity for a small sample size, not all LMICs will be represented in this study, however an economic and geographic spread is sought. We anticipate developing considerable insight from our international cohort of participants with differing levels of income and localities, in addition to establishing an understanding of the ways long-term follow-up of neurotrauma outcomes can be successfully recorded in LMICs. Careful attention to nuances must be paid. We respect and have the foresight that there will be heterogeneity within LMICs as a whole, and even within countries themselves as to the current provision, capabilities and attitudes towards neurotrauma follow-up. We must be sensitive when categorising our findings across a spectrum based on the gross national income per capita. We acknowledge that some false dichotomy may exist in the dialogue regarding healthcare provision, particularly of the collection of long-term outcomes, and caution will be exercised in interpretation of our findings. Unfortunately, due to the resources available and the need to conduct in-depth interviews and subsequent qualitative analysis, it is not possible to recruit non-English speaking physicians.

Furthermore, we recognise that this study may present a narrow perspective on long-term follow-up by only presenting the views of neurotrauma physicians. Given the dearth of literature in this field this study will still make a valuable contribution to the evidence base. However, future studies are encouraged that are inclusive of professional groups such as nurses, allied health professionals and data managers/epidemiologists who may have a wider view on neurotrauma follow-up in LMICs.

Notwithstanding, to our knowledge this study is among the first to examine the contextual challenges associated with long-term follow-up of patients following traumatic brain injury in LMICs. Findings generated from this study will enable us to establish an understanding of the ways long-term follow-up of neurotrauma outcomes can be successfully recorded in LMICs. This knowledge may lend itself to facilitating the ongoing development of cost-effective and novel follow-up technologies in the future.

Author Contributions

The concept of this study was conceived by AGK and PJH as part of Theme 3D of the NIHR Global Health Research, with further study design by CW, AK and BS. CW wrote the first draft of the protocol, with BS preparing the protocol for publication. TB, INE, CK, MK, DJFS, AMR and AF suggested amendments to the protocol and edited the final manuscript.

Competing Interests Statement

Angelos Kolias and Peter Hutchinson are supported by the National Institute for Health Research (NIHR) Cambridge Biomedical Research Centre and the NIHR Global Health Research Group on Neurotrauma. Peter Hutchinson is also supported by a NIHR Research Professorship. The NIHR Global Health Research Group on Neurotrauma was commissioned by the United Kingdom NIHR using Official Development Assistance funding

For peer review only

(project no. 16/137/105). The views expressed in this manuscript are those of the authors and are not necessarily those of the United Kingdom National Health Service, NIHR, or the Department of Health. Drs Esene, Karekezi, Khan, Solla and Kolias are members of the Young Neurosurgeons committee of the World Federation of Neurosurgical Societies. The committee is supporting this project.

Funding Statement
This research was supported by National Institute for Health Research (NIHR) Global Health Research Group on Neurotrauma (grant number 16/137/105) using UK aid from the UK Government.

Data Availability Statement
No additional data are available in this protocol.

References
1 Kolias AG, Rubiano AM, Figaji A, *et al.* Traumatic brain injury: global collaboration for a global challenge. *Lancet Neurol* 2019;18:136–7. doi:10.1016/S1474-4422(18)30494-0
2 De Silva MJ, Roberts I, Perel P, *et al.* Patient outcome after traumatic brain injury in high-, middle- and low-income countries: analysis of data on 8927 patients in 46 countries. *Int J Epidemiol* 2009;38:452–8. doi:10.1093/ije/dyn189
3 Dewan MC, Rattani A, Gupta S, *et al.* Estimating the global incidence of traumatic brain injury. *J Neurosurg* 2019;130:1080–97. doi:10.3171/2017.10.JNS17352
4 Iaccarino C, Carretta A, Nicolosi F, *et al.* Epidemiology of severe traumatic brain injury. *J Neurosurg Sci* 2018;62.
5 Haagsma JA, James SL, Castle CD, *et al.* Burden of injury along the development spectrum: associations between the Socio-demographic Index and disability-adjusted life year estimates from the Global Burden of Disease Study 2017. *Inj Prev* 2020;26:i12–26. doi:10.1136/injuryprev-2019-043296
6 Mock C, Lormand J, Goosen J, *et al.* Guidelines for essential trauma care. Geneva, World Health Organization: 2004.
7 Norton R, Kobusingye O. Injuries. *N Engl J Med* 2013;368:1723–30. doi:10.1056/NEJMra1109343
8 Rubiano AM, Carney N, Chesnut R, *et al.* Global neurotrauma research challenges and opportunities. *Nature* 2015;527:S193–7. doi:10.1038/nature16035
9 Servadei F, Tropeano MP, Spaggiari R, *et al.* Footprint of Reports From Low- and Low- to Middle-Income Countries in the Neurosurgical Data: A Study From 2015 to 2017. *World Neurosurg* 2019;130:e822–30. doi:10.1016/j.wneu.2019.06.230
10 Laytin AD, Seyoum N, Azazh A, *et al.* Feasibility of telephone-administered interviews to evaluate long-term outcomes of trauma patients in urban Ethiopia. *Trauma Surg Acute Care Open* 2018;3:e000256. doi:10.1136/tsaco-2018-000256
11 Laytin AD, Debebe F. The burden of injury in low-income and middle-income countries: knowing what we know, recognising what we don't know. *Emerg Med J* 2019;:emermed-2019-208514. doi:10.1136/emermed-2019-208514
12 Mowafi H, Ngaruiya C, O'Reilly G, *et al.* Emergency care surveillance and emergency care registries in low-income and middle-income countries: conceptual challenges and future directions for research. *BMJ Glob Heal* 2019;4:e001442. doi:10.1136/bmjgh-2019-001442
13 Vaca SD, Xu LW, Nalwanga J, *et al.* Long-term follow-up of pediatric head trauma patients treated at Mulago National Referral Hospital in Uganda. *J Neurosurg Pediatr* 2018;23:125–32. doi:https://dx.doi.org/10.3171/2018.7.PEDS17601
14 Åhman S, Saveman B, Styrke J, *et al.* Long-term follow-up of patients with mild traumatic brain injury: A mixed-method study. *J Rehabil Med* 2013;45:758–64. doi:10.2340/16501977-1182
15 Ruet A, Bayen E, Jourdan C, *et al.* A Detailed Overview of Long-Term Outcomes in Severe Traumatic Brain Injury Eight Years Post-injury. *Front Neurol* 2019;10. doi:10.3389/fneur.2019.00120
16 LaGrone LN, Romaní Pozo DA, Figueroa JF, *et al.* Status of trauma quality improvement programs in the Andean region: What foundation do we have to build on. *Injury* 2017;48:1985–93. doi:10.1016/j.injury.2017.03.003
17 Vaismoradi M, Turunen H, Bondas T. Content analysis and thematic analysis: Implications for conducting a qualitative descriptive study. *Nurs Health Sci* 2013;15:398–405. doi:10.1111/nhs.12048
18 Smith J, Bekker H, Cheater F. Theoretical versus pragmatic design in qualitative research. *Nurse Res* 2011;18:39–51. doi:10.7748/nr2011.01.18.2.39.c8283
19 Bowen GA. Naturalistic inquiry and the saturation concept: a research note. *Qual Res* 2008;8:137–52.

For peer review only

doi:10.1177/1468794107085301
20 Bradshaw C, Atkinson S, Doody O. Employing a Qualitative Description Approach in Health Care Research. *Glob Qual Nurs Res* 2017;4:233339361774228. doi:10.1177/2333393617742282
21 Sullivan-Bolyai S, Bova C, Harper D. Developing and refining interventions in persons with health disparities: The use of Qualitative Description. *Nurs Outlook* 2005;53:127–33. doi:10.1016/j.outlook.2005.03.005
22 Levers M-JD. Philosophical Paradigms, Grounded Theory, and Perspectives on Emergence. *SAGE Open* 2013;3:215824401351724. doi:10.1177/2158244013517243
23 World Bank. World Bank List of Economies 2019. 2019.
24 Moser A, Korstjens I. Series: Practical guidance to qualitative research. Part 3: Sampling, data collection and analysis. *Eur J Gen Pract* 2018;24:9–18. doi:10.1080/13814788.2017.1375091
25 Polit D, Beck C. *Resource Manual for Nursing Research: Generating and Assessing Evidence for Nursing Practice*. 9th ed. Lippincott Williams & Wilkins 2011.
26 Braun V, Clarke V. To saturate or not to saturate? Questioning data saturation as a useful concept for thematic analysis and sample-size rationales. *Qual Res Sport Exerc Heal* 2019;:1–16. doi:10.1080/2159676X.2019.1704846
27 Braun V, Clarke V. What can “thematic analysis” offer health and wellbeing researchers? *Int J Qual Stud Health Well-being* 2014;9:26152. doi:10.3402/qhw.v9.26152
28 Ulin PR, Robinson ET, Tolley EE, *et al. Qualitative Methods in Public Health: A Field Guide for Applied Research*. 2nd ed. 2016.
29 Braun V, Clarke V. Reflecting on reflexive thematic analysis. *Qual Res Sport Exerc Heal* 2019;11:589–97. doi:10.1080/2159676X.2019.1628806
30 Korstjens I, Moser A. Series: Practical guidance to qualitative research. Part 4: Trustworthiness and publishing. *Eur J Gen Pract* 2018;24:120–4. doi:10.1080/13814788.2017.1375092
31 Butler-Kisber L. *Qualitative Inquiry: Narrative and Arts-Informed Perspectives*. 1st ed. Los Angeles: : SAGE 2010.
32 Nicholls D. Qualitative research. Part 3: Methods. *Int J Ther Rehabil* 2017;24:114–21. doi:10.12968/ijtr.2017.24.3.114
33 Lincoln YS, Guba EG. *Naturalistic Inquiry*. 1st ed. California: : SAGE 1985.
34 Birt L, Scott S, Cavers D, *et al.* Member Checking: A Tool to Enhance Trustworthiness or Merely a Nod to Validation? *Qual Health Res* 2016;26:1802–11. doi:10.1177/1049732316654870

For peer review only

BMJ Open

Neurotrauma physicians' perspectives on the contextual challenges associated with Long-term follow-up following Traumatic Brain Injury in Low- and Middle-income countries: a qualitative study protocol

Journal:	*BMJ Open*
Manuscript ID	bmjopen-2020-041442.R3
Article Type:	Protocol
Date Submitted by the Author:	28-Jan-2021
Complete List of Authors:	Smith, Brandon; Addenbrooke's Hospital, Department of Clinical Neurosciences, Division of Neurosurgery; University of Cambridge, NIHR Global Health Research Group on Neurotrauma<br>Whiffin, Charlotte; University of Derby, College of Health of Social Care; University of Cambridge, NIHR Global Health Research Group on Neurotrauma<br>Esene, Ignatius ; University of Bamenda, Neurosurgery Division, Faculty of Health Sciences<br>Karekezi, Claire; Rwanda Military Hospital, Department of Neurosurgery<br>Bashford, Tom; University of Cambridge, NIHR Global Health Research Group on Neurotrauma<br>Mukhtar Khan, Muhammad; Northwest General Hospital & Research Center, Neurosurgery; University of Cambridge, NIHR Global Health Research Group on Neurotrauma<br>Fontoura Solla, Davi; University of São Paulo Department of Neurosciences and Behaviour Sciences; University of Cambridge, NIHR Global Health Research Group on Neurotrauma<br>Indira Devi, Bhagavatula; National Institute of Mental Health and Neuro Sciences; University of Cambridge, NIHR Global Health Research Group on Neurotrauma<br>Hutchinson, Peter; Addenbrooke's Hospital, Department of Clinical Neurosciences, Division of Neurosurgery; University of Cambridge, NIHR Global Health Research Group on Neurotrauma<br>Kolias, Angelos; Addenbrooke's Hospital, Department of Clinical Neurosciences, Division of Neurosurgery; University of Cambridge, NIHR Global Health Research Group on Neurotrauma<br>Figaji, Anthony; University of Cape Town; University of Cambridge, NIHR Global Health Research Group on Neurotrauma<br>Rubiano, Andres M; Universidad El Bosque, Neurosciences Institute, Department of Neurosurgery; University of Cambridge, NIHR Global Health Research Group on Neurotrauma
**Primary Subject Heading**:	Neurology
Secondary Subject Heading:	Global health, Surgery, Qualitative research
Keywords:	QUALITATIVE RESEARCH, NEUROSURGERY, Neurological injury <

	NEUROLOGY

I, the Submitting Author has the right to grant and does grant on behalf of all authors of the Work (as defined in the below author licence), an exclusive licence and/or a non-exclusive licence for contributions from authors who are: i) UK Crown employees; ii) where BMJ has agreed a CC-BY licence shall apply, and/or iii) in accordance with the terms applicable for US Federal Government officers or employees acting as part of their official duties; on a worldwide, perpetual, irrevocable, royalty-free basis to BMJ Publishing Group Ltd ("BMJ") its licensees and where the relevant Journal is co-owned by BMJ to the co-owners of the Journal, to publish the Work in this journal and any other BMJ products and to exploit all rights, as set out in our licence.

The Submitting Author accepts and understands that any supply made under these terms is made by BMJ to the Submitting Author unless you are acting as an employee on behalf of your employer or a postgraduate student of an affiliated institution which is paying any applicable article publishing charge ("APC") for Open Access articles. Where the Submitting Author wishes to make the Work available on an Open Access basis (and intends to pay the relevant APC), the terms of reuse of such Open Access shall be governed by a Creative Commons licence – details of these licences and which Creative Commons licence will apply to this Work are set out in our licence referred to above.

Other than as permitted in any relevant BMJ Author's Self Archiving Policies, I confirm this Work has not been accepted for publication elsewhere, is not being considered for publication elsewhere and does not duplicate material already published. I confirm all authors consent to publication of this Work and authorise the granting of this licence.

For peer review only

Title page

Full title: Neurotrauma physicians' perspectives on the contextual challenges associated with Long-term follow-up following Traumatic Brain Injury in Low- and Middle-income countries: a qualitative study protocol
Short title: Long-term follow-up of TBI in LMICs

Brandon G Smith* [1,2], Charlotte J Whiffin* [1,2,3], Ignatius N Esene[4], Claire Karekezi[5], Tom Bashford[2], Muhammad Mukhtar Khan[2,6], Davi Jorge Fontoura Solla[2,7], Bhagavatula Indira Devi[2,8], Peter J Hutchinson[1,2], Angelos G Kolias[1,2], Anthony Figaji[2,9], Andres M Rubiano[2,10]

* joint first authors

[1] Division of Neurosurgery, Department of Clinical Neurosciences, Addenbrooke's Hospital and University of Cambridge, Cambridge, U.K.
[2] NIHR Global Health Research Group on Neurotrauma, University of Cambridge, Cambridge, UK
[3] College of Health and Social Care, University of Derby, U.K.
[4] Neurosurgery Division, Faculty of Health Sciences, University of Bamenda, Cameroon
[5] Rwanda Military Hospital, Department of Neurosurgery, Rwanda
[6] Northwest School of Medicine & Northwest general Hospital & Research Centre, Peshawar, Pakistan
[7] University of São Paulo, Brazil
[8] Department of Neurosurgery, NIMHANS, Bangalore, India
[9] University of Cape Town, Cape Town, South Africa
[10] Institute of Neurosciences and Neurosurgery, El Bosque University, Bogotá, Colombia

Corresponding author
Angelos G. Kolias
Clinical Lecturer in Neurosurgery
Division of Neurosurgery
Box 167
University of Cambridge
Cambridge Biomedical Campus
Cambridge, CB2 0QQ,
United Kingdom

Email address: ak721@cam.ac.uk

For peer review only

For peer review only - http://bmjopen.bmj.com/site/about/guidelines.xhtml

ABSTRACT

Introduction

Traumatic Brain Injury is a global public health concern; however, low- and middle-income countries (LMICs) face the greatest burden. The World Health Organisation recognise the significant differences between patient outcomes following injuries in high-income countries versus those in LMICs. Outcome data is not reliably recorded in LMICs and despite improved injury surveillance data, data on disability and long-term functional outcomes remains poorly recorded. Therefore, the full picture of outcome post-TBI in LMICs is largely unknown.

Methods and Analysis

This is a cross-sectional pragmatic qualitative study using individual semi-structured interviews with physicians who have experience of neurotrauma in LMICs. The aim of this study is to understand the contextual challenges associated with long-term follow-up of patients following traumatic brain injury in LMICs. For the purpose of the study, we define 'Long-term' as any data collected following discharge from hospital. We aim to conduct individual semi-structured interviews with 24 to 48 neurosurgeons, beginning February 2020. Interviews will be recorded and transcribed verbatim. A reflexive thematic analysis will be conducted supported by NVIVO software.

Ethics and Dissemination

The University of Cambridge Psychology Research Ethics Committee approved this study in February 2020. Ethical issues within this study include consent, confidentiality and anonymity, and data protection. Participants will provide informed consent and their contributions will be kept confidential. Participants will be free to withdraw at any time without penalty; however, their interview data can only be withdrawn up to one week after data collection. Findings generated from the study will be shared with relevant stakeholders such as the World Federation of Neurosurgical Societies and disseminated in conference presentations and journal publications.

Registration*: Not applicable.*

ARTICLE SUMMARY

Study Strengths and Limitations

- This study is among the first to examine the contextual challenges associated with long-term follow-up of patients following traumatic brain injury in LMICs.
- Findings generated from this study will enable us to establish an understanding of the ways long-term follow-up of neurotrauma outcomes can be successfully recorded in LMICs, and may facilitate the ongoing development of cost effective, novel follow-up technologies.
- Through our non-probability sampling technique and necessity for a small sample size, not all LMICs will be represented in this study, however an economic and geographic spread is sought.
- Due to the resources available and the need to conduct in-depth interviews and subsequent qualitative analysis, it was not possible to recruit non-English speaking physicians.

Key Words:

- Traumatic Brain Injury
- Qualitative Research
- Global Neurosurgery
- Follow-up

***Word count:* 3862**

For peer review only

INTRODUCTION

Background

Traumatic Brain Injury (TBI) is a global public health concern; however, low- and middle-income countries (LMICs) face the greatest burden, where 85% of the world's population live[1,2]. Each year, an estimated 69 million cases of TBI are reported globally[3], with a substantial portion of these cases from falls and road traffic incidents [3,4]. In 2017, there were 521 million cases of all non-fatal injuries, a sharp increase from 354 million reported in 1990. When considering disability-adjusted life years (DALYs) from these injuries, road incidents and falls were found to be the main contributors. Haagsma et al. demonstrate that globally, age-standardised DALY rates increase with decreasing Socio-demographic Index (SDI) [5]. The World Health Organisation recognise the significant differences between patient outcomes following injuries in high-income countries (HICs) versus those in LMICs[6], and that almost 90% of deaths occurring from injuries occur in LMICs[7,8]. In high-income countries improvements in surgical interventions and medical management has meant rapid increases in survival rates and improved outcomes. In LMICs fewer resources, different treatment practices and reduced availability of surgical interventions has meant these countries have not all seen the same improvements in outcome post-TBI[9]. Despite a lack of robust data in the literature base to accurately be able to describe the incidence and mortality rates from TBI in LMICs, proportionally, LMICs experience three times as many cases of TBI when compared to HICs[3].

Acknowledging heterogeneity within both LMICs and HICs, as a whole outcome data is generally not reliably recorded in LMICs, and despite improved injury surveillance data, data on disability and long-term functional outcomes remains poorly recorded[10]. Therefore, the full picture of outcome post-TBI in LMICs is largely unknown. Laytin et al (2018) described the difficulty of collecting outcome data in resource poor settings, specifically Sub-Saharan Africa. Collection of outcome data in these settings was typically conducted within the emergency department or at hospital discharge rather than at points after discharge. Challenges of collecting outcome data included weak healthcare infrastructure and limited regular follow-up of trauma patients[10].

Despite such challenges the collection of long-term outcomes is essential, as it provides epidemiological data to properly determine the full burden of injury and assess the efficacy of patient treatment and management decisions in LMICs[10,11] In addition Mock et al.,[6] argues that to fully understand the full burden of disease, disability must be evaluated as well as death. The collection of long-term outcomes is additionally important in the implementation and continuation of registries. These resources serve to provide opportunities for continuous quality improvement and enable trials of interventions in these populations – the results of these registries can themselves be incorporated into legislation and specific care pathways[12]. Therefore, ways must be found to collect more complex long-term outcomes following TBI in LMICs that are directly comparable to the outcome data being collected in most high-income countries. Further adding to this challenge is a lack of clarity in the global definition of 'long-term' in neurotrauma, and what period this includes; a study of long-term outcomes after paediatric head trauma in Uganda defined long-term as anything 1-2 years post-injury[13], whilst studies from France and Sweden suggest long-term as periods spanning at least 3 years and beyond[14,15].

In turn, we must be mindful of the complex relationship between the economic status of each country and the quality of their neurosurgical follow-up provision. Many LMICs have highly heterogeneous health systems, and countries with a low gross national income per capita may still support well-functioning neurosurgical centres offering a high level of follow-up care. Although, on average, poorer countries might be expected to demonstrate worse follow-up as a function of resource limitation, we must be careful not to assume an association at the hospital level.

Rationale

This study is the first in a three-phase project which aims to explore long-term follow-up in LMICs, its challenges, facilitators and technological solutions to augment current practices. The existing literature appears to support an association between the utilisation of trauma registries, quality improvement programmes, and improved long-term outcomes. This association has been used to suggest a role for systems science approaches to improving neurotrauma care, particularly in LMICs[8,12,16]. A preliminary review of the evidence base revealed how little empirical literature had been published about the difficulties long-term follow-up poses within LMICs, and what specific contextual challenges exist within these countries. Without an in-depth understanding of these contextual challenges it was not possible to begin work on technological solutions to improve the

For peer review only - http://bmjopen.bmj.com/site/about/guidelines.xhtml

collection of long-term outcomes in these countries. Therefore, an exploratory qualitative study was designed to provide an in-depth understanding of neurotrauma physicians' attitudes, experiences, and perspectives of follow-up. For the purpose of this study a 'neurotrauma physician' is defined as any medical doctor or surgeon directly involved in the provision of acute neurotrauma care. This foundation of understanding will make a meaningful contribution to the limited empirical evidence base while also informing later phases of the project examining technological solutions in low resources settings using quantitative methods.

This project is part of the NIHR Global Health Group on Neurotrauma and contributes to its strategic aims. The NIHR Global Health Group on Neurotrauma commenced in 2017 aiming to understand current systems and challenges in TBI clinical care within LMICs and as part of this acknowledged that the collection of long-term outcomes needed to be improved. Although beyond the scope of this particular project, results of this study may warrant an investigation into how well long-term follow-up is conducted across the spectrum of high-income country neurosurgical centres, that are not directly conducting or engaging in long-term outcome studies for objective comparison. In addition, we will be careful to ensure our results are not compared to presumptions, or to centres with follow-up efforts motivated only by engagement in international studies.

Theoretical Framework

This study uses a qualitative methodology which is especially useful to explore complex phenomena[17] when little is known about a topic or where new insights are required. Qualitative inquiry is committed to subjectivity and the collection of rich in-depth data through the examination of participant's viewpoints[17]. Pragmatic qualitative research, also known as descriptive and generic qualitative research, does not align itself to any specific philosophical perspective and therefore allows flexibility of methods that enables the study to be designed in an appropriate way to address the research question[18]. Pragmatic research lies within the naturalistic paradigm which recognises the existence of multiple realities and studies real-world situations by having little impact on the phenomena under investigation[19]. Pragmatic qualitative studies aim to achieve in-depth understanding prioritising literal description first and then understanding phenomena in a deeper sense through analysis and interpretation of how people draw meaning from their experiences[20]. The goal of such research is to provide an account of the "experiences, events and process that most people (researchers and participants) would agree are accurate"[21] [p128]. In addition, Sullivan-Bolyai, et al.[21] state qualitative descriptive designs are particularly useful when researching populations of other cultures and as such would be well suited to the aims of this study.

Research Aim

The aim of this study is to understand the contextual challenges associated with long-term follow-up of patients following traumatic brain injury in LMICs. For the purpose of the study we define 'Long-term' as any data collected following discharge from hospital.

Objectives

1. To determine current approaches to long-term follow up of patients following TBI in LMICs
2. To explore the challenges associated with long-term follow-up of patients following TBI in LMICs.
3. To collaboratively offer recommendations for appropriate solutions that will facilitate long-term follow-up of patients following TBI in LMICs based on findings and themes identified

METHODS AND ANALYSIS

Study Design

We propose a cross-sectional pragmatic qualitative study using individual semi-structured interviews with physicians who have experience of neurotrauma in LMICs. Despite the pragmatic nature of this study, the naturalistic paradigm in which it is situated aims to interpret individual experiences and employs a subjective epistemology which asserts universal knowledge is not possible[22]. A purposive sampling strategy will be employed to recruit 24 - 48 participants from LMIC countries participating in the NIHR GHRGN. Methods include semi-structured telephone/online video interviews and the collection of relevant demographic and professional data.

Data collection

This study will collect data through semi-structured telephone or online video interviews, conducted by a single interviewer (BS). Semi-structured interviews are the cornerstone of qualitative methodology as they facilitate

For peer review only - http://bmjopen.bmj.com/site/about/guidelines.xhtml

rich data gathering and in-depth exploration of the phenomenon under investigation. As such semi-structured interviews were the natural choice for data collection in this study. Telephone and online video methods were chosen over face-to-face for pragmatic reasons given the geographic location of the participants and the interviewer. We will also collect some limited demographic data that will help us analyse and interpret the similarities and differences between the data sets (See Box One).

Specific Demographic data will be collected using an electronic form (see Box One).

Age<br>Sex<br>Country of residence<br>Occupation<br>Years of experience<br>Years working in neurotrauma<br>Practice setting
Box One: Demographic Data

Details of the interview questions are presented in Box Two, and participants will be made aware of these prior to the interview so they have the opportunity to consider their response. Questions are open ended to encourage a rich dialogue and participants will be free to elaborate on their answers adding additional detail as required. To encourage more in-depth responses prompts, probes and follow-up questions will be used. In this sense the questions in Box Two will be used more as a guide than an explicit framework, which is consistent with a semi-structured approach. It is anticipated that the interviews will last up to 60 minutes and will be conducted by BS following a pilot of the interview guide. Interviews will be recorded using a digital recorder.

Current follow-up

A. Please can you tell me about how and when your patients are currently followed-up following discharge from a TBI?
    a. What outcomes are measured?
    b. What needs are assessed?
    c. What referrals are made?
    d. What type of contact system do you have in place (face-to-face, telephone, telemedicine)?

Definition and understanding of long-term outcomes

A. What do you understand by the use of 'long- term' in the context of outcomes post-TBI?
B. Do you think your definition and understanding of 'long-term outcomes' in your country are the same as those in other countries?
C. Do you know or use any type(s) of outcome classification for follow-up?

Attitudes towards long-term follow-up

A. What do you think about the need for long-term follow-up of patients following TBI in your setting?
B. What would you consider to be the main benefits of long-term follow-up of TBI patients?
C. What do you consider to be the challenges associated with long-term follow-up of your patients with TBI?
D. Are these challenges more related to: health system aspects, national, regional, or local administrative aspects, institutional aspects, resources for care aspects?
E. Are there any other issues that need to be considered for long-term follow-up in your own setting and wider country?

Long-term follow-up in LMIC

A. Thinking more broadly now about other Low- and Middle-Income Countries, do you have any other thoughts about long-term follow-up in these other LMICs
B. What else might be required in LMICs to facilitate long-term follow-up of patients following TBI?

Possible interventions to facilitate follow-up in LMIC

A. Do you know what types of technology patients and families have access to that could help in follow-up of patients following TBI?
B. What kind of technology would help in your country to record long-term follow-up?
C. Are you aware of any specific mobile health, telemedicine, or phone interview options for long-term follow-up in your institution/state/country?

For peer review only - http://bmjopen.bmj.com/site/about/guidelines.xhtml

D. Does your service or institution participate in a trauma or neurotrauma quality improvement program including the use of clinical registries with outcome measures?
Box Two: Interview guide

Study Setting

Participants will be recruited from any country identified as low or middle income as defined by the 2017-2018 World Bank list of economies[23]. Access to participants will be negotiated using the research group's project lead and collaborators from the twelve countries in the NIHR Global Health Research Group on Neurotrauma listed in Box Three. New collaborators in the group, including the Philippines and Zimbabwe, in addition to participants from institutions from LMICs not yet part of the group, will be invited to participate as necessary in order to attain the defined sample size.

1. BRAZIL	7. MYANMAR
2. COLOMBIA	8. NIGERIA
3. ETHIOPIA	9. PAKISTAN
4. INDIA	10. SOUTH AFRICA
5. INDONESIA	11. TANZANIA
6. MALAYSIA	12. ZAMBIA
Box Three: LMICs in the NIHR Global Health Research Group on Neurotrauma	

Eligibility Criteria

To be eligible for this study the person must be a practising physician within an LMIC and have experience of managing neurotrauma, so they have experience of treatment and discharge of patients with TBI. We are requesting the physicians all have at least two years' experience of neurotrauma and also some experience of research, so they have more insight into the complexities of long-term follow-up of neurotrauma patients and understand the research process. Due to availability of resources participants will have to be fluent in English. Experience of working with collaborators would suggest this criterion will not limit participation in the study. All participants will have to provide written informed consent by agreeing to the criteria within the electronic consent form.

Inclusion criteria

• Physician within a LMIC with experience of managing neurotrauma<br>• At least two years' experience of neurotrauma<br>• Experience of collecting, or attempting to collect, TBI outcome data<br>• Self-declared fluency in spoken English<br>• Able to provide informed consent
Box Four: Inclusion criteria

Sample

The main criticism of qualitative studies stems from the necessity for small samples. Smaller samples facilitate depth of inquiry and rich interpretation. However, according to Sullivan-Bolyai, et al.[21] qualitative descriptive studies can accommodate more moderate sample sizes than theoretically informed studies because there is no requirement to generate theory. Therefore Sullivan-Bolyai, et al.[21] recommends a sample size of 20-50 participants.

Moser and Korstjens[24] explain that a guiding principle of sampling in qualitative studies is to sample up until data saturation is reached. Data saturation is a qualitative principle which is a point whereby the researchers are confident that new data will not reveal new information[25]. However, data saturation is a contentious issue in reflexive thematic analysis which advocates a less prescriptive approach to sampling and asks the researcher to make an interpretive judgement about when to stop[26].

For peer review only - http://bmjopen.bmj.com/site/about/guidelines.xhtml
For peer review only

In line with this view, sample size in this study was further informed by a desire to explore a variety of views from physicians within a range of LMICs. A non-probability, purposive sample is therefore required. We will aim to have representation from a range of countries and, if possible, a range of regions within these countries. Therefore, we propose a sample size of 24 - 48 participants and will comment further on principles of data saturation in the final paper.

Recruitment

We will begin by seeking to recruit 2-3 neurotrauma physicians from the collaborating countries in the NIHR Global Health Research Group on Neurotrauma, consisting of 12 LMICs (Box Three), with new collaborators from Zimbabwe and the Philippines. We will also be placing a call through social media (Twitter and WhatsApp) to maximise the opportunity for recruitment from any physicians not subscribed to the GHRGN mailing list or from other LMICs not listed as collaborating regions.

The local primary investigators (PIs) will be written to through the group's previously established mailing lists, with information pertaining to the study including the aims of the study, inclusion criteria (Box Four) for participants and data collection methods. We will then ask the local PIs to forward information about the study to relevant physicians (NIHR GHRGN collaborators are also eligible to participate). For social media recruitment an infographic was developed for the study and distributed through Twitter as an open call for participants. Those who wished to express an interest can contact the lead author (BS) for more information. Information forwarded to participants will include a letter of invitation, participant information sheet, and contact details for the research team. If interested in participating an initial telephone call will be arranged to review the participant information sheet and the requirements of the study. If the physician still wishes to continue, we will schedule a telephone interview. We will leave a minimum of one week in-between this initial contact and arrangement of the interview to allow a cooling off period. If an insufficient number of people have responded to the first call for participants a second request for participation will be made two – three weeks later. Data will be collected over a 12-month period, beginning March 2020.

It is essential that participants join this study of their own free will. However, the notion that junior colleagues may have a 'reciprocal obligation' to assist their more senior colleagues, and the institution, in participating in global research is appreciated. To safeguard against potential coercion, we will highlight to our participants that they are under no obligation to take part. We will discuss this specifically during the pre-consent meeting and ensure they understand that no-one will be informed if they chose, or do not chose, to participate in the study. Participants will not be provided with any financial reimbursement, as no out of pocket expenses are anticipated.

Data Analysis

Analysis within pragmatic qualitative studies should stay close to the text, and does not require "highly abstract rendering of data"[20]. Staying close to the text means early coding decisions rely heavily on what participants actually said with interpretation applied more substantially in later phases of analysis. Studies which are independent of theory, can and often do use thematic analysis[17,27]. According to Braun and Clarke[27] thematic analysis can reveal a rich, detailed and complex exploration of the data and despite its 'descriptive' title will end with some necessary interpretation of the data[20] through discovery of shared and varied patterns of understanding[28]. Indeed Vaismoradi, et al.[17] contends that thematic approaches actually attends little to the description of the data set and instead requires interpretation of the various aspects of the phenomena under investigation. It is therefore proposed that a six stage Braun and Clarke[27] thematic analysis will be conducted (See Box Five). Congruent with Braun and Clarke's more recent commentary on thematic analysis, this study aligns itself to an approach described as 'reflexive TA'[29].

To complete the analysis audio files from interviews will first be transcribed verbatim by a transcription service and checked for accuracy by BS. Codes will be assigned to individual transcripts and will be supported by the use of NVIVO software, allowing researchers to organise the data, share coding decisions and confirm the origins of interpretation. Similar codes will be grouped together, and initial themes identified. Themes will then be reviewed, revised and final themes named and agreed upon within the research team. BS will take lead on the data analysis, supported by CW in the initial coding stages. This process of coding and theme building is not about reaching consensus between coders about the way codes were applied or their interpretation[29]. Instead, this is a collaborative process to advance understanding of the data and develop a more nuanced and

For peer review only - http://bmjopen.bmj.com/site/about/guidelines.xhtml

reflexive interpretation congruent with our epistemological position. We will then report our final interpretive themes and the supporting sub-themes identified in the analysis. These will be defended through critical discussion and the origins of the themes evidenced through the presentation of anonymised direct quotes. A more in-depth discussion of the data analysis process and audit trail will be presented in the findings paper.

BS is a PhD student and fourth year medical student who has undertaken training in qualitative methods and analysis. CW is an experienced qualitative researcher and nurse academic.

1. Familiarising yourself with your data<br>2. Generate initial codes<br>3. Searching for themes<br>4. Reviewing themes<br>5. Defining and naming themes<br>6. Produce the report
Box Five: Braun and Clarke [27] thematic analysis framework

Rigour

Validity, generalizability and reliability have little relevance in qualitative research[30,31]. Instead qualitative research is judged by more appropriate criteria such as credibility and trustworthiness[32]. These hallmarks of quality determine the rigour of a qualitative study. In this study we intend to include a number of strategies advocated by Nicholls[32] to increase the rigour of the methods and consequently the final findings. First, following a reflexive, interpretive approach our findings will be strengthened through the critical dialogue between members of the research team, principally BS and CW, and subsequently with our co-authors. In addition, we propose using member checking as advocated by Lincoln and Guba[33] as crucial to establishing credibility in qualitative studies and according to Birt, et al.[34] can overcome issues of researcher bias and increases transferability of findings. In this regard, we will first return any transcripts where transcription was not possible due to the quality of recording to ask for clarification of specific sections of the interview if necessary. Second, we will share initial themes identified from the data with participants so they can add further insight to the interpretation of findings if they wish. These contributions will be added to the final analysis of data.

ETHICS & DISSEMINATION

This is an open, online study with global participants, with the primary investigator and principal research team all based in the UK. Although including overseas respondents, as this research is hosted and delivered by the UK, favourable opinion is only sought from a UK ethics committee.

Subsequently, in February 2020 The University of Cambridge Psychology Research Ethics Committee reviewed this study and provided favourable ethical opinion (Ref: PRE.2020.010), and thus is in line with the Concordat to Support Research Integrity from Universities UK, Research Councils UK and the UK Research Integrity Office (2013), International Ethical Guidelines for Health-related Research Involving Humans, Council for International Organizations of Medical Sciences (CIOMS) 2016, The World Medical Association's (WMA) Declaration of Helsinki and Good Clinical Practice (the ICH GCP R2 2016) as applicable. The University of Cambridge is the sponsor and appropriate insurance is in place. Ethical issues within this study include consent, confidentiality and anonymity, and data protection.

All participants will be required to provide written informed consent after a cooling-off period of at least a week after the participant information sheet has been delivered. Participants can withdraw at any time without penalty, with a grace period of a week given after completion of the interview to withdraw their interview data, after which withdrawal of data will not be possible as analysis will have commenced.

All information will be kept strictly confidential, with all information generated to be stored on a GDPR compliant confidential server. A unique study ID will be allocated to all participants and used to anonymise personal data. Audio files will be immediately transferred to an encrypted folder and the data on the recording device destroyed, interview data will be anonymised upon transcription and other personal information changed or

For peer review only - http://bmjopen.bmj.com/site/about/guidelines.xhtml

removed once checked by the research team. Participants will be told that anonymised quotes will be published in the findings of this study; however, despite this anonymity it is possible that their contributions may be recognisable to others reading the study. We will also be seeking consent to disclose region and level of income associated with their country of origin against participant quotes used in the publication of findings, as this will be important to the contextual understanding of the study.

Patient & Public Involvement

This study seeks to ascertain the views of physicians, and thus patient and public involvement was not considered to be as relevant in this context. However, we recognise the value of working with key stakeholders to develop research and therefore asked for peer review of this study by collaborating members of the GHRGN. Their comments informed the final study design.

Dissemination

When we have completed the study, we will produce a study summary which we will offer a copy to participants via email. Study findings will also be disseminated internationally through the GHRGN network and other relevant stakeholders such as the World Federation of Neurosurgical Societies, in addition to appropriate conferences, peer reviewed journal publications and social media.

Study Limitations

Understandably, through the non-probability approach to sampling and the necessity for a small sample size, not all LMICs will be represented in this study, however an economic and geographic spread is sought. We anticipate developing considerable insight from our international cohort of participants with differing levels of income and localities, in addition to establishing an understanding of the ways long-term follow-up of neurotrauma outcomes can be successfully recorded in LMICs. Careful attention to nuances must be paid. We respect and have the foresight that there will be heterogeneity within LMICs as a whole, and even within countries themselves as to the current provision, capabilities and attitudes towards neurotrauma follow-up. We must be sensitive when categorising our findings across a spectrum based on the gross national income per capita. We acknowledge that some false dichotomy may exist in the dialogue regarding healthcare provision, particularly of the collection of long-term outcomes, and caution will be exercised in interpretation of our findings. Unfortunately, due to the resources available and the need to conduct in-depth interviews and subsequent qualitative analysis, it is not possible to recruit non-English speaking physicians.

Furthermore, we recognise that this study may present a narrow perspective on long-term follow-up by only presenting the views of neurotrauma physicians. Given the dearth of literature in this field this study will still make a valuable contribution to the evidence base. However, future studies are encouraged that are inclusive of professional groups such as nurses, allied health professionals and data managers/epidemiologists who may have a wider view on neurotrauma follow-up in LMICs.

Notwithstanding, to our knowledge this study is among the first to examine the contextual challenges associated with long-term follow-up of patients following traumatic brain injury in LMICs. Findings generated from this study will enable us to establish an understanding of the ways long-term follow-up of neurotrauma outcomes can be successfully recorded in LMICs. This knowledge may lend itself to facilitating the ongoing development of cost-effective and novel follow-up technologies in the future.

Author Contributions

The concept of this study was conceived by AGK and PJH as part of Theme 3D of the NIHR Global Health Research, with further study design by CW, AGK and BS. CW wrote the first draft of the protocol, with BS preparing the protocol for publication. TB, INE, CK, MK, BID, DJFS, AMR and AF suggested amendments to the protocol and edited the final manuscript.

Competing Interests Statement

Angelos Kolias and Peter Hutchinson are supported by the National Institute for Health Research (NIHR) Cambridge Biomedical Research Centre and the NIHR Global Health Research Group on Neurotrauma. Peter Hutchinson is also supported by a NIHR Research Professorship. The NIHR Global Health Research Group on Neurotrauma was commissioned by the United Kingdom NIHR using Official Development Assistance funding

For peer review only - http://bmjopen.bmj.com/site/about/guidelines.xhtml

(project no. 16/137/105). The views expressed in this manuscript are those of the authors and are not necessarily those of the United Kingdom National Health Service, NIHR, or the Department of Health. Drs Esene, Karekezi, Khan, Solla and Kolias are members of the Young Neurosurgeons committee of the World Federation of Neurosurgical Societies. The committee is supporting this project.

Funding Statement
This research was supported by National Institute for Health Research (NIHR) Global Health Research Group on Neurotrauma (grant number 16/137/105) using UK aid from the UK Government.

Data Availability Statement
No additional data are available in this protocol.

References

1 Kolias AG, Rubiano AM, Figaji A, *et al.* Traumatic brain injury: global collaboration for a global challenge. *Lancet Neurol* 2019;18:136–7. doi:10.1016/S1474-4422(18)30494-0
2 De Silva MJ, Roberts I, Perel P, *et al.* Patient outcome after traumatic brain injury in high-, middle- and low-income countries: analysis of data on 8927 patients in 46 countries. *Int J Epidemiol* 2009;38:452–8. doi:10.1093/ije/dyn189
3 Dewan MC, Rattani A, Gupta S, *et al.* Estimating the global incidence of traumatic brain injury. *J Neurosurg* 2019;130:1080–97. doi:10.3171/2017.10.JNS17352
4 Iaccarino C, Carretta A, Nicolosi F, *et al.* Epidemiology of severe traumatic brain injury. *J Neurosurg Sci* 2018;62.
5 Haagsma JA, James SL, Castle CD, *et al.* Burden of injury along the development spectrum: associations between the Socio-demographic Index and disability-adjusted life year estimates from the Global Burden of Disease Study 2017. *Inj Prev* 2020;26:i12–26. doi:10.1136/injuryprev-2019-043296
6 Mock C, Lormand J, Goosen J, *et al.* Guidelines for essential trauma care. Geneva, World Health Organization: 2004.
7 Norton R, Kobusingye O. Injuries. *N Engl J Med* 2013;368:1723–30. doi:10.1056/NEJMra1109343
8 Rubiano AM, Carney N, Chesnut R, *et al.* Global neurotrauma research challenges and opportunities. *Nature* 2015;527:S193–7. doi:10.1038/nature16035
9 Servadei F, Tropeano MP, Spaggiari R, *et al.* Footprint of Reports From Low- and Low- to Middle-Income Countries in the Neurosurgical Data: A Study From 2015 to 2017. *World Neurosurg* 2019;130:e822–30. doi:10.1016/j.wneu.2019.06.230
10 Laytin AD, Seyoum N, Azazh A, *et al.* Feasibility of telephone-administered interviews to evaluate long-term outcomes of trauma patients in urban Ethiopia. *Trauma Surg Acute Care Open* 2018;3:e000256. doi:10.1136/tsaco-2018-000256
11 Laytin AD, Debebe F. The burden of injury in low-income and middle-income countries: knowing what we know, recognising what we don't know. *Emerg Med J* 2019;:emermed-2019-208514. doi:10.1136/emermed-2019-208514
12 Mowafi H, Ngaruiya C, O'Reilly G, *et al.* Emergency care surveillance and emergency care registries in low-income and middle-income countries: conceptual challenges and future directions for research. *BMJ Glob Heal* 2019;4:e001442. doi:10.1136/bmjgh-2019-001442
13 Vaca SD, Xu LW, Nalwanga J, *et al.* Long-term follow-up of pediatric head trauma patients treated at Mulago National Referral Hospital in Uganda. *J Neurosurg Pediatr* 2018;23:125–32. doi:https://dx.doi.org/10.3171/2018.7.PEDS17601
14 Åhman S, Saveman B, Styrke J, *et al.* Long-term follow-up of patients with mild traumatic brain injury: A mixed-method study. *J Rehabil Med* 2013;45:758–64. doi:10.2340/16501977-1182
15 Ruet A, Bayen E, Jourdan C, *et al.* A Detailed Overview of Long-Term Outcomes in Severe Traumatic Brain Injury Eight Years Post-injury. *Front Neurol* 2019;10. doi:10.3389/fneur.2019.00120
16 LaGrone LN, Romaní Pozo DA, Figueroa JF, *et al.* Status of trauma quality improvement programs in the Andean region: What foundation do we have to build on. *Injury* 2017;48:1985–93. doi:10.1016/j.injury.2017.03.003
17 Vaismoradi M, Turunen H, Bondas T. Content analysis and thematic analysis: Implications for conducting a qualitative descriptive study. *Nurs Health Sci* 2013;15:398–405. doi:10.1111/nhs.12048
18 Smith J, Bekker H, Cheater F. Theoretical versus pragmatic design in qualitative research. *Nurse Res* 2011;18:39–51. doi:10.7748/nr2011.01.18.2.39.c8283
19 Bowen GA. Naturalistic inquiry and the saturation concept: a research note. *Qual Res* 2008;8:137–52.

For peer review only - http://bmjopen.bmj.com/site/about/guidelines.xhtml

doi:10.1177/1468794107085301
20 Bradshaw C, Atkinson S, Doody O. Employing a Qualitative Description Approach in Health Care Research. *Glob Qual Nurs Res* 2017;4:233339361774228. doi:10.1177/2333393617742282
21 Sullivan-Bolyai S, Bova C, Harper D. Developing and refining interventions in persons with health disparities: The use of Qualitative Description. *Nurs Outlook* 2005;53:127–33. doi:10.1016/j.outlook.2005.03.005
22 Levers M-JD. Philosophical Paradigms, Grounded Theory, and Perspectives on Emergence. *SAGE Open* 2013;3:215824401351724. doi:10.1177/2158244013517243
23 World Bank. World Bank List of Economies 2019. 2019.
24 Moser A, Korstjens I. Series: Practical guidance to qualitative research. Part 3: Sampling, data collection and analysis. *Eur J Gen Pract* 2018;24:9–18. doi:10.1080/13814788.2017.1375091
25 Polit D, Beck C. *Resource Manual for Nursing Research: Generating and Assessing Evidence for Nursing Practice*. 9th ed. Lippincott Williams & Wilkins 2011.
26 Braun V, Clarke V. To saturate or not to saturate? Questioning data saturation as a useful concept for thematic analysis and sample-size rationales. *Qual Res Sport Exerc Heal* 2019;:1–16. doi:10.1080/2159676X.2019.1704846
27 Braun V, Clarke V. What can “thematic analysis” offer health and wellbeing researchers? *Int J Qual Stud Health Well-being* 2014;9:26152. doi:10.3402/qhw.v9.26152
28 Ulin PR, Robinson ET, Tolley EE, *et al. Qualitative Methods in Public Health: A Field Guide for Applied Research*. 2nd ed. 2016.
29 Braun V, Clarke V. Reflecting on reflexive thematic analysis. *Qual Res Sport Exerc Heal* 2019;11:589–97. doi:10.1080/2159676X.2019.1628806
30 Korstjens I, Moser A. Series: Practical guidance to qualitative research. Part 4: Trustworthiness and publishing. *Eur J Gen Pract* 2018;24:120–4. doi:10.1080/13814788.2017.1375092
31 Butler-Kisber L. *Qualitative Inquiry: Narrative and Arts-Informed Perspectives*. 1st ed. Los Angeles: : SAGE 2010.
32 Nicholls D. Qualitative research. Part 3: Methods. *Int J Ther Rehabil* 2017;24:114–21. doi:10.12968/ijtr.2017.24.3.114
33 Lincoln YS, Guba EG. *Naturalistic Inquiry*. 1st ed. California: : SAGE 1985.
34 Birt L, Scott S, Cavers D, *et al.* Member Checking: A Tool to Enhance Trustworthiness or Merely a Nod to Validation? *Qual Health Res* 2016;26:1802–11. doi:10.1177/1049732316654870

For peer review only
